# Targeting the CCL2/CCR2 Axis in Cancer Immunotherapy: One Stone, Three Birds?

**DOI:** 10.3389/fimmu.2021.771210

**Published:** 2021-11-03

**Authors:** Liyang Fei, Xiaochen Ren, Haijia Yu, Yifan Zhan

**Affiliations:** Department of Drug Discovery, Shanghai Huaota Biopharm, Shanghai, China

**Keywords:** CCL2, CCR2, cancer immunotherapy, macrophages, T regulatory cells

## Abstract

CCR2 is predominantly expressed by monocytes/macrophages with strong proinflammatory functions, prompting the development of CCR2 antagonists to dampen unwanted immune responses in inflammatory and autoimmune diseases. Paradoxically, CCR2-expressing monocytes/macrophages, particularly in tumor microenvironments, can be strongly immunosuppressive. Thus, targeting the recruitment of immunosuppressive monocytes/macrophages to tumors by CCR2 antagonism has recently been investigated as a strategy to modify the tumor microenvironment and enhance anti-tumor immunity. We present here that beneficial effects of CCR2 antagonism in the tumor setting extend beyond blocking chemotaxis of suppressive myeloid cells. Signaling within the CCL2/CCR2 axis shows underappreciated effects on myeloid cell survival and function polarization. Apart from myeloid cells, T cells are also known to express CCR2. Nevertheless, tissue homing of Treg cells among T cell populations is preferentially affected by CCR2 deficiency. Further, CCR2 signaling also directly enhances Treg functional potency. Thus, although Tregs are not the sole type of T cells expressing CCR2, the net outcome of CCR2 antagonism in T cells favors the anti-tumor arm of immune responses. Finally, the CCL2/CCR2 axis directly contributes to survival/growth and invasion/metastasis of many types of tumors bearing CCR2. Together, CCR2 links to two main types of suppressive immune cells by multiple mechanisms. Such a CCR2-assoicated immunosuppressive network is further entangled with paracrine and autocrine CCR2 signaling of tumor cells. Strategies to target CCL2/CCR2 axis as cancer therapy in the view of three types of CCR2-expessing cells in tumor microenvironment are discussed.

## Introduction

A broad array of chemokines and chemokine receptors regulate physiological and pathological processes, including tumorigenesis (1). CC-chemokine receptor 2 (CCR2) is highly expressed by a subset of Ly6Chi monocytes with strong proinflammatory functions (2). CCR2 deficiency markedly reduces Ly6Chi monocytes trafficking out of bone marrow and to sites of inflammation (3–5). CCR2 deficiency also reduces Th1 response and the severity of experimental autoimmune diseases (6, 7). Similarly, deletion of CCR2+ monocytes has a profound impact on immunity to infection and autoimmunity (8, 9). Despite this, CCR2 antagonism as a treatment for autoimmune diseases has been met with the disappointing results (10). Further, CCR2 antagonism has been found to exacerbate autoimmune diseases, suggesting an opposite immune regulatory role for CCR2, although the basis of anti-inflammatory role of CCR2 remains undefined (11). As the role of CCR2 in regulation of autoimmunity remains contradictory, overwhelming evidence supports that the CCL2/CCR2 axis activity largely favors progression and metastasis of tumor by attracting suppressive monocytes and Tregs (12, 13), though any chemokine/chemokine receptor can have both pro-tumor and anti-tumor action (1). Beyond chemotaxis, CCR2 can also directly impact the function of myeloid cells and T cells in a less defined fashion. This review will separately discuss the chemotactic and non-chemotactic effects of the CCL2/CCR2 axis on monocytes/macrophages, T cells and tumor cells. We surmise that two main types of suppressive immune cells (e.g. monocytic myeloid suppressors and Treg cells) in suppressive tumor microenvironments, are more dependent on CCR2 signaling and thus the net outcome of CCR2 signaling favors tumor progression and metastasis. Together with the paracrine and autocrine CCR2 signaling of tumor cells, CCR2 has a significant role in tumor growth and metastasis. Thus, targeting the CCL2/CCR2 axis may be a plausible avenue in cancer therapy, particularly for many solid tumors belonging to the “cold tumor” family.

## CCR2 Ligands and CCR2 Signaling

Since being reported in 1994 (14), CCR2 is the second most studied chemokine receptor after CCR5 (based on PubMed) and continues to be actively investigated as a potential drug target for many diseases, ranging from autoimmune diseases, diabetes and chronic pain syndromes, to atherosclerosis, HIV and cancer. Over 2 decades, structure, expression, expression regulation of CCR2 and its ligands, and CCR2 signaling has been detailed (15). Here, we provide brief discussion on this aspect of CCL2/CCR2 biology, as more detailed dissection of cell type specific expression of and functions of CCL2/CCR2 is covered in corresponding sections of this review.

CCR2 belongs to the chemokine receptor subfamily of human Class A G protein-coupled receptors (GPCRs). In humans, two isoforms CCR2A and CCR2B differ in their C-terminal which can result in different signaling properties. CCR2 is known to be expressed by monocytes/macrophages. Consequently, deficiency grossly affects traffic of monocytes/macrophages (3, 6). Nevertheless, CCR2 is also expressed by various cell types including Tregs (16), CD4^+^ T cells (17). CD8^+^ T cells (18); NKTs (19), γδT cells (20), B cells (21), plasmacytoid dendritic cells (22), basophils (23), stem cells (24), endothelial cells (25), microglia (26), muscle cells (27) and tumor cells (28). Expression of CCR2 is subject to regulation by many various factors. CCR2 expression by monocytes can be upregulated by plasma cholesterol, peroxisome proliferator-activated receptor gamma ligands and salt (29, 30). On the other hand, hypoxia-induced HMGB1 downregulates CCR2 expression by monocytes (31). CCR2 on human NK cells can also be induced by IL-2 (32).

CCL2 is the prototype chemokine binding to CCR2, and the CCL2-CCR2 pairing is the most relevant for CCR2 function. Nevertheless, CCR2 can be activated by other chemokines including CCL7 (MCP-3) (33, 34), CCL8 (MCP-2) (35), CCL12 (MCP-5), CCL13 (MCP-4) (36), CCL16 (37). In general, chemokines other than CCL2 have been less explored for their contribution to CCR2-mediated function.

**Figure 1:**
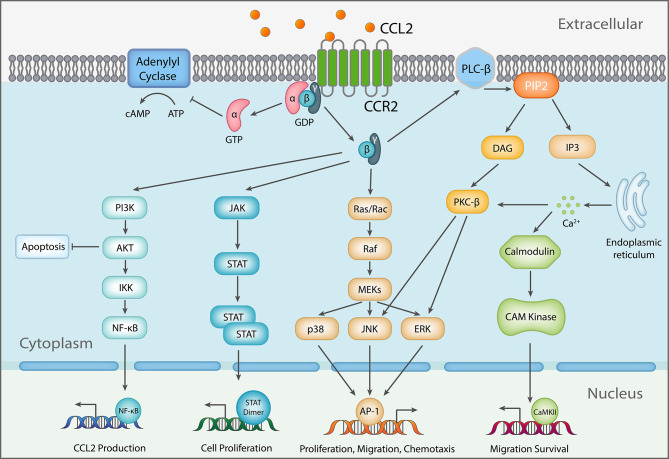
Schematic diagram of CCL2 signaling. As a response to CCL2 binding at the N-terminus, extracellular loops and transmembrane bundle of CCR2, the intracellular G-protein αi subunit dissociates from the CCR2 and the βγ subunit. The α subunit then inhibits adenylyl cyclase (AC) function resulting in decreased cyclic adenosine monophosphate levels. In contrast, the βγ subunit signaling induces gene expression *via* several pathways, further inducing changes to cellular function [Adapted from (38)].

Ligation of the chemokine receptor CCR2 leads to activation of multiple downstream signaling pathways (**Figure 1**). The CCR2-mediated signal transduction starts with CCL2/CCR2 binding and activation of GPCR (39) and then activates PI3K/Akt pathway (40–42), RAC GTPase pathway (43), PKC-dependent pathway (44), and JAK/STAT pathway. Conceivably, activation of different pathways affects different biological processes, ranging from cell survival, proliferation, migration, and differentiation. The downstream activation of PI3K/Akt pathway protects tumor cells from death and promotes proliferation (45, 46). Activated PI3K induces activation of protein kinase B (Akt) *via* phosphorylation at Thr308 and Ser473 by PDK1 and PDK2, respectively (40, 41, 47), which in turn mediates up-regulation of survivin and down-regulation of autophagosome formation *via* promoting mammalian target of rapamycin (mTOR) activation (42, 48). Thereafter, the crucial survival protein, survivin, inhibits two major programmed cell death pathways – apoptosis (49–51) and autophagic death (52). Consequently, it allows tumor cells, such as PC3 and VCaP prostate cancer cells, to survive from cell death stimuli, like nutrition starvation (42). In addition, it has been shown that PI3K/Akt plays a central role in chemotaxis by inducing IKKα β phosphorylation, which in turn increases NF-kB transactivation and consequently promotes MMP-9 expression (53–55). MMP-9 aids in cell migration through degrading the extracellular matrix (ECM) (56), while CCL2 induces migration of other CCR2-expressing cells. Activation of downstream MEK/ERK pathway (57) can result in up-regulation of gene expression, such as MMP-9 (58), which promotes migration. Additionally, PKC is activated as a downstream signal of G-protein dissociation, and prompts activation of JNK and ERK to promote cancer cell migration (59, 60). CCL2/CCR2 signaling also triggers the JAK/STAT pathway by activating Janus kinase 2 (JAK2) (61), and thereby triggers downstream pathways, including STAT1, STAT3, and STAT5 (62, 63), which further inhibit apoptosis and induce extravasation and expansion of tumor cells like colon carcinoma (64).

As mentioned above human CCR2 has two forms: CCR2A and CCR2B. They differ only in their terminal carboxyl tails and cellular location (14, 65). We discuss here some differences in expression and function which may have implications in inflammation and cancer immunity. CCR2B was the predominant isoform a mRNA level in human monocytes (65). In some pathological conditions, the CCR2 show cell specific expression (66). For example, mononuclear cells in disease lesions show strong expression of CCR2A but not CCR2B (66). Further, CCR2 isoforms shows preferential induction by inflammatory stimulation (67). Exposure of fibroblast like synoviocytes of patients with RA to sCD40L caused strong upregulation of CCR2A but not of CCR2B protein expression. CCR2A is also notably frequently overexpressed in glioblastoma (68). In stable transfected Jurkat cells, CCR2A and CCR2B show some subtle difference in CCL2-eminated signaling (69). CCL2 induced a transient Ca(2+) flux in the CCR2B transfected cells but not in the CCR2A transfected cells. Together, CCR2A and CCR2B may have differences in their function and distribution.

## Chemotactic and Non-Chemotactic Effects of CCL2/CCR2 Axis On Monocytes/Macrophages

### Functional Conundrum of CCR2-Expressing Monocytes/Macrophages: Inflammatory or Suppressive?

Monocytes consist of different subsets and are the prime source of tissue macrophages. The dominant subset of mouse CD11b^+^Ly6C^+^ monocytes and human CD14^+^ monocytes express CCR2. Early work has established that CCR2 expressing mouse Ly6C^hi^ monocytes are potent producers of proinflammatory cytokines (2). Consequently, deletion of CCR2^+^ monocytes/macrophages profoundly reduces immunity to infection and autoimmunity (8). However, MØs do not always function as proinflammatory and immuno-stimulatory. Rather, they have a broad spectrum of function that depends on their surrounding environmental cues. Particularly, many soluble factors modulate MØ function and their corresponding molecular programming. Interferon (IFN)-γ/LPS has been found to induce proinflammatory M1 MØs with a potent ability to produce cytokines priming Th1, Th17 and CTL response while IL-4 induces alternatively activated M2 MØs with a more immunosuppressive role (70). Many other cytokines also influence MØ functional polarization. For example, GM-CSF and M-CSF have been shown to generate distinct MØ populations with certain M1 and M2 characteristics from mouse bone marrow (BM) precursors (71). Despite GM-CSF being considered a M1 MØ inducer *in vitro* and *in vivo* for both mouse and human MØs (9, 71–75), high levels of GM-CSF have also been associated with development of suppressive M2-like MØs (76, 77). Of note, pre-treatment of MØs with CCL2 selectively inhibited IL-12 production in response to IFN-γ and Staphylococcus aureus (78).

In the context of cancer, MØs also function in a spectrum from tumoricidal, immuno-stimulating to immunosuppressive. MØs within the local tumor microenvironment (TME) generally polarize to be immunosuppressive. There are two terms “Tumor associated macrophages (TAM)” and monocytic myeloid derived suppressor cells (M-MDSCs) to describe closely connected monocytic myeloid cells in TMEs (79, 80). M-MDSCs are present in blood and lymphoid organs and defined as CD11b^+^Ly6C^+^Ly6G^-^. They develop towards TAM that express lower levels of Ly6C and higher levels of MHCII, F4/80 and CX3CR1 (79). Abundant evidence shows that TAMs are derived from Ly6C^+^CCR2^+^ monocytes in various experimental tumor models (81–85). Due to the heterogenous nature of TMEs, factors that polarize TAMs into suppressive M2 like MØs are also likely heterogenous. Consequently, functional programs of TAMs can also be versatile, ranging from cancer initiation, angiogenesis, immune suppression to tumor metastasis (86). Given their CCR2^+^ cell origin, the CCL2/CCR2 axis is critically for TAM development, *via* chemotactic as well as non-chemotactic mechanisms.

### The Role of CCL2/CCR2 Axis in Monocyte Recruitment

Early work with CCR2 deficient mice firmly establishes a role for CCR2 in monocyte traffic and recruitment (6, 87). Firstly, CCR2 contributes to monocyte egress from bone marrow, leading to monocytopenia in the absence of CCR2 (87). Secondly, CCR2 is critically required for influx of inflammatory monocytes/MØs in respond to innate inflammatory stimulation (6). Results from adoptive transfer of WT and CCR2-/- monocytes also support that CCR2 is required for migration of monocytes from the blood to inflamed tissues (87). Impairments in monocyte migration have also been reported in CCL2-/- mice (88). Interestingly, deficiency of another CCR2 ligand MCP-3 but not MCP-2 and MCP-5 also profoundly reduces monocyte egress from bone marrow (87).

TAMs can be derived from both resident MØs and monocyte-derived MØs (84, 85). CCR2 clearly contributes to the influx of monocytes/MØs to tumors, particularly metastatic sites to generate TAMs (12, 80, 84, 89). In a MMTV-PyMT (PyMT) mammary tumor model (84), depletion of tumor-associated monocytes significantly reduced TAMs. Of note, compared to resident mammary tissue MØs, TAMs had a higher proliferative capacity (84). Further, TAM populations in this model expressed Vcam1 but did not express typical M2 markers such as Ym1, Fizz1, and Mrc1 (84). In a gliomas model, CCR2 deficiency also reduced CD11b^+^/Ly6C^hi^/PD-L1^+^ MDSCs and inhibition of CCR2 increased median survival (90). Fittingly, a combination of CCR2 inhibition and anti-PD-1 increased survival of glioma-bearing mice (90).

CCR2 mediated recruitment of blood monocytes or M-MDSCs to tumor tissues results in a high abundance of TAMs that is often associated with poor clinical outcomes in patients. In human breast cancer, the level of CCL2, produced by monocytic cells and tumor cells, was associated significantly with TAM accumulation and was a significant indicator of early relapse (91) and vessel invasion of tumor cells (92). However, in another study with breast carcinoma, CCL2 expression was not found to be closely correlated with MØ infiltrations, although carcinomas showed higher levels of CCL2 mRNA than normal breast tissue (93). In esophageal carcinogenesis, high expression of CCL2 also correlated with TAM accumulation and was associated with poor prognosis (94). Overall, there is support that recruitment of TAM by CCL2 has clinical significance.

### The Role of CCL2/CCR2 Axis in Survival/Proliferation and Functional Polarization of Monocytes/Macrophages

As the chemotactic role of CCL2/CCR2 axis is well-established, the non-chemotactic role of CCL2/CCR2 axis is less understood but has been receiving recent attention (95). CCL2/CCR2 signaling can directly impact on survival/proliferation, functional polarization, effector molecule secretion, autophagy, killing, and adhesion of monocytes/MØs (95). We briefly discuss here the non-chemotactic roles of the CCL2/CCR2 axis: survival/proliferation and functional polarization of monocytes/MØs, particularly in the context of TAMs.

Human monocytes can be separated into CD14^+^CD16^−^ classical monocytes, CD14^+^CD16^+^ intermediate monocytes, and CD14^lo^CD16^+^ nonclassical monocytes. Classical monocytes have a very short circulating lifespan (about 1 d). Most classical monocytes leave the circulation or die, and the remaining cells become intermediate monocytes. Intermediate monocytes have a longer lifespan (about 4 days) before transitioning to nonclassical monocytes. Nonclassical monocytes have the longest lifespan in the blood, of around 7 days, before either leaving the circulation or dying (96). Similarly, mouse monocyte subsets follow a similar pattern (97).

Homeostasis of immune cells is critically regulated by the BCL-2 regulated apoptosis pathway. Several pro-survival members of the BCL-2 family including BCL-2, BCL-xL, A1, MCL-1, and BCL-w promote survival of immune cells (98). However, survival of monocytes/macrophages at steady state is not essentially affected by loss/inhibition of BCL2 (99), BCL-xL (100, 101), MCL-1 (102, 103) or A1 (104). CCL2 induces upregulation of Bcl-2, Bcl-XL and cFLIP and protects monocytes from apoptosis under serum deprivation stress (105). During inflammation, CCL2 also linked to MØ survival (106). Nevertheless, current knowledge is still very limited regarding survival control of TAMs and the contribution of axis of CCL2/CCR2 to survival of monocytes/TAMs. As TAMs replenishment requires the continuous recruitment of M-MDSC and monocytes, TAMs also expressed high levels of Ki67 staining and EdU incorporation, indicating cell proliferation and self-renewal (84). CCL2, as well as CCL3 and CCL14 can induce MØ proliferation (107).

CCL2 also seems to affect functional polarization of MØs. Related to the suppressive nature of TAMs and M-MDSCs, CCL2 has been found to induce human CD206^+^ MØs (105). Inclusion of CCL2 in monocytes cultured with M-CSF or GM-CSF also increased M2 markers *in vitro* (108). Pre-treatment of MØs with CCL2 selectively inhibited IL-12 production in response to IFN-γ and Staphylococcus aureus (78). Therefore, CCL2/CCR2 signaling axis can promote the polarization of M2 like MØs. However, it is somewhat unclear how CCL2 induces M2 polarization in the above tumor context. Notably in certain conditions, deficiency of CCL2 or CCR2 biases for M2 polarization (109, 110), highlighting the complexity of functional polarization of MØs. Overall, tumor microenvironment contains many factors favoring the development of M2 like TAMs, CCL2/CCR2 signaling axis not only play a major role in recruitment of monocytes/MØs to contribute to functional polarization of MØs but also can directly polarize MØs. The significance of the direct signaling to functional polarization of MØs remains to be further explored.

### The Role of CCL2/CCR2 Axis in Monocytes/MØs-Mediated Tumor Metastasis

Contribution of CCR2-mediated recruitment of monocytes in cancer metastasis is nicely demonstrated in a study with monocyte transfer (12). Adoptive transfer of Ly6C^hi^CD11b^+^ cells from wt but not CCR2-/- mice resulted in preferential recruitment of monocytes to a tumor cell challenged lung (12). Transfer of human CCR2-expressing CD14^+^ monocytes into CSF-1 supplemented nude mice with inoculation of human MDA-MB-231-derived metastatic breast cancer cells also led to selective tumor recruitment of CD14^+^ monocytes to lung (12). Neutralization of CCL2 or CCR2 deficiency blocks monocyte recruitment, reduces MØs interacting with tumor cells and more importantly reduces lung metastasis (12). Notably, monocyte-derived VEGF has a role in promoting tumor extravasation (12). Fittingly, CCL2 induced chemotaxis of human endothelial cells and induced the formation of blood vessels *in vivo* (12). Neutralization of CCL2 also inhibits chemotaxis of human endothelial cells, increases survival and inhibits lung metastases in mice bearing human breast carcinoma cells (111).Tumor metastasis involves epithelial-mesenchymal transition (EMT). At molecular level, a group of matrix metalloproteinases (MMPs) plays a critical role in the process. Cells of the monocyte/MØ lineage secrete diverse MMPs in large quantities, stimulated by many cellular and soluble factors (112). Conceivably, CCL2/CCR2 signaling recruits abundant TAMs to produce MMPs in response to intra-tumoral environmental cues. Nevertheless, direct CCR2 signaling on macrophages also modulates MMP production (113).

## CCR2 on T Cells Mediates Chemotactic and Non-Chemotactic Functions

### CCR2 Is Expressed by T Cell Subsets and Contributes to Tissue Homing of T Cells

It has been known for a long time that the chemotactic axis of the CCL2/CCR2 also directly contributes to T cell chemotaxis (114). Phenotyping of chemoattracted T lymphocytes shows that CCR2-expressing T cells have an activated/memory phenotype (114). Further, Th1 cells have been shown to have higher expression of CCR2 and are more responsive to CCL2 induced migration (17). Activated but not naïve CD8^+^ T cells during viral infection also express CCR2 (18). Of note, CCR2 expression by T cells also shows location and disease preference (115). CCR2^+^ lymphocytes, predominantly CD4^+^ T cells but not CD8^+^ T cells, are increased in ileal Crohn’s disease (CD), but not colonic CD or in ulcerative colitis. Such an increase in CCR2^+^ lymphocytes in ileal CD is limited to lesions, without any increase in circulating CCR2^+^ T lymphocytes (115). Functionally, experiments with CCR2-/- mice provided the evidence that CCR2 deficiency greatly impacts on Th1 and Th2 responses (6, 116, 117). However, in most cases, the contributions of CCR2 to myeloid cells and T cells in induction of a T cell response has not been delineated.

Regulatory T cells (Treg) also express CCR2 (16, 118). Notably, FoxP3^+^ cells, particularly CD62L^-^ ones, expressed higher levels of CCR2 than FoxP3^-^ T cells (16). Fittingly, Ag-primed FoxP3^+^ T cells migrate more efficiently to nonlymphoid tissues than FoxP3^−^ T cells, although it is not clear from the study whether CCR2 mediates the migration (16). In mixed chimera mice containing both WT and CCR2-/- cells (controlling for differences in myeloid cell counts), there was a profound and selective defective accumulation of CCR2-/- FoxP3^+^ cells in liver, lung and adipose tissue (119, 120). In an allograft model with Treg transfer, CCR2-/- Tregs migrate poorly to the inflamed grafts. Consequently, transfer of CCR2-/- Tregs did not improve allograft survival (118). High levels of CCR2 ligand also results in preferential local accumulation of Tregs (121). Overall, Tregs, compared to conventional T cells, seem to more rely on CCR2 for migration to tissue.

### CCR2 Directly Regulates Function of Conventional T Cells and Tregs

There is also *in vitro* and *in vivo* evidence supporting non-chemotactic roles for CCR2 in regulation of T cell differentiation. When CD4^+^ T cells were activated either by antigen-pulsed APCs or polyclonal stimuli in the presence of CCL2 *in vitro*, T cells showed an increase in production of IL-4, but not IFN-γ (122), implying a role independent of chemotaxis. In order to investigate the intrinsic activity of CCR2 on T cells independent of the innate cellular microenvironment, naïve CD45RB^hi^ CD4^+^, CD45RB^lo^ effector/memory CD4^+^ or CD25^+^ Tregs from WT and CCR2-/- mice were transferred into immunodeficient host RAG1-/- mice (123). Mice transferred with naïve or effector CD4^+^ cells from WT but not CCR2-/- donors caused significant weight loss in recipient mice. However, the numbers of donor T cells accumulated in the colon were not significantly different between WT and CCR2-/- cells (123). Rather, CCR2-/- T cells showed defective production of IFN-γ and IL-17 and were biased towards Treg differentiation. In support of an early *in vitro* study (122), CCR2-/- CD4^+^ T cells cross-linked using anti-CD3/CD28 produced less cytokines IL-17F, IL-22, IFN-γ, and IL-10 without impacting on cell proliferation and CCL2 seems to mediate the effect (123). This also occurs under Th17 and Th1 polarizing conditions. In contrast, CCR2-/- CD4^+^ T cells in the presence of TGFβ differentiated more efficiently into Foxp3^+^ Tregs (123). At a signaling level, the levels of p-Akt were significantly reduced in CCR2-/- T cells following T cell activation. Although GPCR chemokine receptors induce activation of the PI3K/Akt network (**Figure 1**), it is not clear from the study what the up-stream signals leading to CCR2-mediated PI3K/Akt activation are.

Similar to conventional T cells, CCR2 may also directly impact on Treg function. *In vivo*, CCR2-/- Tregs are less capable of suppressing alloimmunity compared to WT Tregs when they are directly transferred into graft tissue (118). Although CCR2 deficiency does not grossly affect accumulation of Tregs in lymphoid organs (118, 119), we found that Foxp3^+^ CD4^+^ T cells in lymphoid organs and peripheral tissues of CCR2-/- mice express lower levels of CD25 (119), bearing in mind that CD25 expressed by Tregs can serve as an IL-2 sink to execute immune suppression (124). Fittingly, we found that CCR2-/- Tregs *in vitro* were less effective at suppressing T cell proliferation. The signaling events mediated by CCR2 leading to CD25 upregulation are currently unclear. *In vitro* innate activation by CpG can lead to CD25 upregulation on FoxP3^+^ cells in a CCR2 dependent manner but CpG did not stimulate the overt upregulation of CD25 when FoxP3^+^ T cells were isolated and cultured alone. Further CCL2 alone could not phenocopy CpG stimulation (119). Of note, in CCR2-DTR mice expressing diphtheria toxin receptor (DTR) under control of the Ccr2 locus, DT (diphtheria toxin) treatment deleted CD11b^+^Ly6C^+^ monocytes/MØs but did not significantly deplete Tregs and conventional T cells, likely due to lower expression level of CCR2 on lymphocytes (119).

Overall, there is evidence supporting the idea that CCR2 directly regulates the function of conventional T cells and Tregs. However, the findings are rather patchy and contradictory. For example, CCR2 deficiency has a negative impact on Treg function in some studies (118, 119), but not others (123). Thus, the significance of this aspect of CCR2 function in the context of chemotactic roles of CCR2 in migration of myeloid cells and T cells remains to be elucidated.

### CCR2 Preferentially Mediates the Recruitment of Tregs to Tumors

CCR2 can conceivably affect homing of different T cell subsets to tumoral tissues. Loyher et al. had comprehensively investigated the role of CCR2 in recruitment of Tregs and conventional T cells to tumor in different tumor models, as well as human oral squamous cell carcinoma (OSCC) (13). In mice injected subcutaneously with a tumor cell line, tumoral accumulation of Tregs but not conventional CD4^+^ CD25^-^Foxp3^-^ T cells was significantly reduced by CCR2 deficiency. The preferential tumor recruitment of Tregs was intrinsic to Tregs since parabiosis experiments between WT and Foxp3-EGFP CCR2-/- mice show that EGFP^+^ CCR2-/- Tregs which migrated to the tumor tissue represented only 20% of total Treg in both WT hosts and CCR2-/- hosts. Live imaging also revealed that 36% WT were highly motile while only 12% CCR2-/- Tregs belonged to the highly motile group. Using CCL2 binding as a way detecting CCR2-expressing cells, tumor Tregs contained >50% CCR2^+^ while proportions of CCR2^+^ Tregs in circulation and draining LNs were much lower (13). Together, the data supports that CCR2 represents a major Treg homing receptor in tumor contexts. Apart from cell homing, CCR2^+^ Tregs had a heightened ability to produce IL-10 and self-renew than CCR2-/- counterparts, and also expressed higher levels of CD39 but not CD73 when tumor Tregs were separated into two cohorts according to their CCR2 expression (13). Interestingly to note, low dose cyclophosphamide has been shown to selectively deplete CCR2^+^ Tregs, probably due to their higher activating and proliferating state (13).

## CCR2 Signaling in Cancerous Cells Promote Survival/Growth and Metastasis

CCL2 can be produced by multiple cell types in the tumor environment, while CCR2 can also be expressed by multiple cell types including tumor cells. As illustrated above, the signaling axis of CCL2/CCR2 in monocytes/macrophages and T cells has a great impact on cancer immunity, and accumulating evidence also supports the concept that cancerous cells can directly employ CCR2 to promote survival/growth and metastasis. Here we provide an expanded discussion on the topic.

### Tumor Cells Express CCR2 and Produce CCL2

Expression of CCR2 by cancer cells is rather widespread, although the levels of expression may be extremely heterogenous. A study on osteosarcoma revealed CCR2 mRNA expression in all samples (125). In renal cell carcinoma, >50% metastatic tumors were CCR2^+^ (126). Not surprisingly, AML patients (65%) also had CCR2 expressing cancer cells (127). Interesting to note, the study did not observe a direct correlation between CCR2 expression by qPCR vs protein expression by western blot or flow cytometry, implying that post-translational events such as protein degradation could contribute to CCR2 regulation. We surveyed the literature for CCR2 expression by various human tumor cell lines as well as patient samples and found that all types of solid and blood cancers expressed either CCR2 (**Table 1**). The human protein atlas database (www.proteinatlas.org) also showed expression of CCR2 mRNA and protein by multiple types of cancers. Of note, CCR2 expression by tumor cells is also subject to regulation by its own ligands, as CCL2 has been shown to downregulate CCR2 in tumor cells (159). CCR2 expression by tumor cells can respond in a paracrine fashion to CCL2 produced by various non-tumor cells, tumor cells can also produce CCL2 which acts on CCR2 on tumor cells *via* an autocrine fashion. Notably, many tumor cell lines also produce CCL2 (**Table 1**). Comparison of cancer and non-cancer tissues indicated that a higher proportion of prostate cancer tissue samples produced CCL2 (160). In most cases, expression of CCR2 or CCL2 predicts metastasis and poor survival. For example, CCR2 mRNA expression correlates with prostate cancer progression and pathologic stages (128, 161).

**Table 1:** Expression of CCR2 and CCL2 by human tumor lines and patient samples.

Type of tumor cell	Cell line	CCR2	CCL2	Reference
**Breast carcinoma**	MDA-MB-231	**+**	**+/-**	(128–130)
	MCF10A	**+**	**-**	(131)
	MCF7	**+**	**+**	(129)
	4T1	**+**	**+**	(128, 129)
	**Patient samples**	**+**	**+**	(132)
**Osteosarcoma**	MG63	**+**	**+**	(133)
	U2OS	**+**	**+**	(133)
	HOS	**+**	**+**	(133)
	**Patient samples**	**+**		(125)
**Non-small cell lung carcinoma**	A549	**+**	**+**	(128, 134)
	NCI-H460	**-**	**+**	(134)
	NCI-H1299	**+**		(128)
	LC99A	**-**	**-**	(134)
	**Patient samples**	**+**	**+**	(135)
**Hepatocellular carcinoma**	Huh-7	**+**	**+**	(136, 137)
	HepG2	**+**	**+**	(136, 138)
	Hep3B,		**+**	(136, 138)
	MHCC-97L,		**+**	(136)
	MHCC-97H	**+**	**+**	(136, 138)
	LM3		**+**	(136)
	SMMC-7721		**-**	(136, 138)
	**Patient samples**	**+**	**+**	(136, 138)
**Oophoroma**	OVCAR-3	**+**		(139)
	SK-OV-3	**+**	**+**	(139, 140)
	**Patient samples**	**+**		(139)
**Gastric adenocarcinoma**	SGC7901		**+**	(141)
	BGC823		**+**	(141)
	GES-1	**Inducible**		(141)
	GC1401, GC1415 and GC1436	**-**		(142)
	**Patient samples**	**+**	**+**	(141, 143)
**Prostatic cancer**	PC-3	**+**	**+**	(28, 128)
	DU145	**+**		(128)
	LNCap	**+**		(128)
	C42B	**+**		(128)
	**Patient samples**	**+**		(128)
**Pancreatic cancer**	Panc-1		**+**	(144)
	PC13		**+**	(144)
	PT45P1		**+**	(144)
	Capan-1		**+**	(144)
	MiaPaca-2		**+**	(144)
	**Patient samples**		**+**	(145)
**Renal carcinoma**	786-O	**+**	**+**	(146)
	CaKi-1	**+**	**+**	(146)
	**Patient samples**	**+**	**+**, CCL7**+**	(126, 147)
**Melanoma/epidermoid carcinoma**	LM16-R cells		**+**	(148, 149)
	A431		**+**	(150)
	**Patient samples**		**+**	(148, 149)
**Glioblastoma/meningioma**	IOMM Lee		**+**	(151)
	SF-3061		**+**	(151)
	**Patient samples**	**+**	**+**	(152, 153)
**Colorectal cancer**	HCT116	**+**		(154)
	SW480	**+**		(154)
	SW1116p21 cells		**+**	(155)
	**Patient samples**	**+**	**+**	(154–156)
**Head and neck cancer**	**Patient samples**	**+**		(157)
**Blood cancer**	Kas	**+/-**		(127, 128)
	Raw264.7	**+**	**+**	(43)
	THP-1	**+**	**+**	(127)
	NB4	**+**	**+**	(158)
	U937	**+/-**	**+**	(127, 159)
	**Patient samples**	**+**		(127)

Another important player in tumor microenvironment is cancer-associated fibroblasts (CAFs) or tumor-associated fibroblasts (TAFs) (162). CAFs can promote cancer progression by multiple mechanisms. One mechanism is to produce CCL2 (163). Interestingly, CAFs from breast cancer patients but not normal human mammary fibroblasts expressed high levels of CCL2 mRNA in response to activation by various breast cancer cells (163). CAF-derived CCL2 could induce cancer stem cells by activating NOTCH signaling. Consequently, knockdown of CCL2 in CAFs inhibits *in vivo* tumorigenesis cells (163). In support, murine fibroblast activation protein (FAP)^+^ CAFs were a major source of CCL2 (164). In the system, FAP activates STAT3–CCL2 signaling cascade to recruit TAMs and promoted tumor growth in a murine liver cancer model. Fittingly, in human intrahepatic cholangiocarcinoma, high expressions of stromal FAP and CCL2 were negatively associated with ICC patient survival (164). In a murine skin cancer model, CAFs also produced CCL2 to retain TAMs and promote skin carcinogenesis (165). In a study with human liver CAFs and cancer lines, CAFs could up-regulate CCL2 expression in cancer cells (166). Thus, CAFs can participate tumor progression and metastasis *via* CCL2/CCR2 axis.

### CCR2 Signaling Promotes Tumor Metastasis/Invasion

In this section, we mainly consider tumor metastasis/invasion from the angle of direct CCR2 signaling of tumor cells. A large body of evidence comes from *in vitro* investigations with tumor cell lines of different types. THP-1 cells are known for high expression of CCR2 and undergo enhanced migration in the presence of CCL2 (127). Such migration was significantly reduced in the presence of a neutralizing CCL2 monoclonal antibody or blocking CCR2 monoclonal antibody. On the other hand, the CCR2^lo^ U-937 cell line showed no CCL2-induced migration (127). Similarly, migration studies with patient AML samples could also be blocked by an Ab to CCR2 or CCR2 inhibitor (127). For solid tumors, CCL-2 enhanced the migration and invasion of MCF-7 cells (128, 167). Migration of RCC cells in the presence of a CCR2 antagonist or neutralizing CCL2 antibody was also markedly decreased compared with control cells and enhanced by supplemented CCL2 (128, 146).

As discussed, MMPs are a family of zinc-dependent endoproteinases and facilitate tumor cell invasion/metastasis. Within the MMPs, MMP-9 has been shown to degrade the extracellular matrix (ECM) to remove physical barriers for metastasis, increase cell motility, and promote angiogenesis (56, 168). *In vitro* studies have shown that CCL2 can increase MMP-9 expression in human chondrosarcoma cells (55), in NSCL A549 cell migration (167) and in human breast cancer MCF-7 cells (169). Upstream signaling leading to MMP-9 upregulation involves activation of protein kinases MEK/ERK and NF-κB (55). Thus, the CCL2/CCR2 axis not only regulates MPP production and therefore tumor invasion/metastasis *via* its action on TAMs, but also *via* its action on tumor cells directly.

### CCR2 Signaling Promotes Tumor Growth/Survival

The direct action of CCR2 on tumor cells can also lead to survival and proliferation of tumor cells. CCL2 has been found to promote survival and proliferation of THP-1 (127), prostate cancer line PC3 cells (43), renal cell carcinoma lines 786-O and CaKi-1 cells and lung carcinoma line A549 cells (134). Accordingly, cells which were grown in serum-free medium and treated with a CCL2 neutralizing antibody or a CCR2 antagonist (RS504393) had significantly decreased proliferation of tumor cells compared with untreated control cells (134, 146). Of note, endogenous CCL2 expression levels appear to be sufficient for the maximum proliferation rate of 786-O and CaKi-1 cells but not for the maximum migratory ability of RCC cells (146). Related to cancer treatment, CCL2 significantly reduced apoptosis of mouse and human breast cancer cells induced by gentamicin or 5-FU treatment as determined by cleaved caspase-3 expression (129). CCR2-/- cancer cells were also more sensitive to CTL-mediated killing, compared to WT cancer cells (170). However, it has also been reported that CCL2 and GM-CSF-CCL2 fusion protein induced BAX expression and apoptosis in CCR2^+^ EG7 but not CCR2^-^ B16 cells (171). At a signaling level, activation of the PI3 kinase/Akt pathway was found to be vital for the CCL2-mediated proliferative effects of prostate cancer epithelial cells (43). Moreover, CCL2 can promote the viability, proliferation and invasiveness of endometrial stromal cells through the Akt and MAPK/Erk1/2 signal pathways (172). However, understanding of cell death pathways influenced by the CCL2/CCR2 is still very limited.

## Development of CCR2 Antagonism in Cancer Therapy: How Do We Get There?

### Clinical and Preclinical Studies Targeting CCL2/CCR2 Axis: An Update

Perhaps owing to the critical role of CCR2 in the traffic of inflammatory monocytes, CCR2 antagonists have been developed by many pharmaceutical companies for potential use in the treatment of rheumatoid arthritis, asthma, diabetes, inflammatory bowel disease and multiple sclerosis, cardiovascular diseases (173) (**Tables 2**, **3**). However, CCR2 antagonists had not generated favorable outcomes in several Phase II clinical studies (173). Redundancy of the target, poor drug-like properties, insufficient drug level and many other factors could all contribute to the failed clinical trials with small molecules targeting chemokine receptors (173).

**Table 2:** Antagonists targeting CCR2-CCL2 axis.

Product	Developer	Stage	Target	Indications	Status	References/Trial ID
AZD2423	AstraZeneca	II	CCR2	Chronic obstructive pulmonary disease (COPD)	Inactive	NCT01215279
BMS-687681	Bristol-Myers Squibb	Pre	CCR2, CCR5	Cancer	Active	(174)
BMS-741672	Bristol-Myers Squibb	II	CCR2	Type II diabetes	Inactive	NCT00699790
BMS-813160	Bristol-Myers Squibb	I/II	CCR2, CCR5	Pancreatic cancer, Colorectal cancer, Liver cancer, NSCL	Active	NCT03184870; NCT04123379
BMS-813160	Bristol-Myers Squibb	II	CCR2, CCR5	Type II diabetes	Active, Inactive	NCT01752985
CCX140	ChemoCentryx, Vifor Pharma	II	CCR2	Type II diabetes, Fibrosis	Active	(175), NCT01447147.
CCX872	ChemoCentryx	I/II	CCR2	Pancreatic cancer	Active, Inactive	NCT02345408, NCT03778879
Cenicriviroc	Takeda Pharmaceuticals, Dong-A Pharma, Tobira Therapeutics (AbbVie)	II	CCR2, CCR5	HIV	Inactive	(176)
Cenicriviroc	Takeda Pharmaceuticals, Dong-A Pharma, Tobira Therapeutics (AbbVie)	III	CCR2, CCR5	NASH, HIV, COVID19	Active, Inactive	NCT03028740
CNTX-6970	Centrexion Therapeutics	I	CCR2	Pain	Active	NCT03787004
INCB8696	Incyte	I	CCR2	MS	Inactive	(177)
INCB3344	Incyte	Pre	CCR2	MS, RA	Inactive	(178)
INCB3284	Incyte	Pre	CCR2	Undefined inflammation	Inactive	(179)
INCB10820	Incyte, Pfizer	Pre	CCR2, CCR5	Autoimmune diseases	Inactive	(180)
JNJ-17166864	Johnson & Johnson	II	CCR2	Allergic rhinitis	Inactive	NCT00604123
JNJ-27141491	Johnson & Johnson	Pre	CCR2	MS	Inactive	(181)
JNJ-41443532	Johnson & Johnson	II	CCR2	Type II diabetes	Inactive	NCT01230749
MK-0812	Merck & Co.	II	CCR2	RA, MS	Inactive	NCT00239655
PF-04136309	Incyte, Pfizer	II	CCR2	Pancreatic cancer, Arthritic pain, Chronic hepatitis	Inactive	NCT02732938; NCT00689273; NCT01226797
PF-04634817	Pfizer	II	CCR2, CCR5	Type II diabetes	Inactive	NCT01712061
RAP-103	Creative Bio-Peptides	Pre	CCR2, CCR5, CCR8	Neurophathic Pain	Active	(182)
RO5234444	Roche	Pre	CCR2	Type II diabetes	Inactive	(183)
SSR150106	Sanofi	II	CCR2	RA	Inactive	NCT00545454
Tropifexor+Cenicriviroc	Allergan (AbbVie), Novartis	II	CCR2, CCR5, FXR	NASH	Active	CLJC242A2201J; NCT03517540
WXSH0213	WuXi AppTec, Zhongsheng Pharmaceuticals	Pre	CCR2, CCR5	NASH	Active	(184)
Bindarit	Angelini	II	CCL2	Type II diabetes, Atherosclerosis	Inactive	NCT01109212
NOX-E36	NOXXON Pharma AG	II	CCL2	Type II diabetes	Inactive	NCT01547897
NOX-E36	NOXXON Pharma AG	Pre	CCL2	Liver fibrosis	Inactive	(185)

**Table 3:** Antibodies targeting CCR2-CCL2 axis.

Product	Developer	Stage	Target	Indications	Status	Reference/Clinical Trail No.
Anti-CCR2	Pfizer, Amgen,	Pre	CCR2	Solid tumor, Inflammation	Active	US9238691B2
Anti-CCR2	MRC, U.Regensburg	Pre	CCR2	MS, RA	Active	US9068002B2
Anti-CCR2	Sorrento	Pre	CCR2	MS	Inactive	(186)
Anti-CCR2, CSF-1R	Elstar	Pre	CCR2, CSF-1R	Inflammation	Not Clear	WO1997031949A1
CCL2-LPM	Osprey Pharmaceuticals	I	CCR2	IgA nephropathy	Inactive	NCT00856674
Plozalizumab (MLN1202)	Takeda Pharmaceuticals	II	CCR2	Solid tumors	Inactive	NCT02723006
Plozalizumab (MLN1202)	Takeda Pharmaceuticals	II	CCR2	Atherosclerosis	Inactive	NCT00715169
VET2-L2 (oncolytic virus)	Astellas Pharma, KaliVir Immunotherapeutics	Pre	CCR2, leptin, IL-2	Solid tumors	Active	(187)
ABN912	Novartis	I	CCL2	RA	Inactive	(188)
Carlumab (CNTO 888)	Johnson & Johnson	II	CCL2	Solid tumor, Prostate cancer	Inactive	NCT01204996, NCT00992186 (189),
Carlumab (CNTO 888)	Johnson & Johnson	II	CCL2	Idiopathic pulmonary fibrosis,	Inactive	NCT00786201
ABN912	Novartis	Pre	CCL2	Tumor	Not Clear	(190)
Anti-CCL2	Shire Human Genetic Therapies	Pre	CCL2	Scleroderma	Not Clear	WO2013177264A1

**Figure 2:**
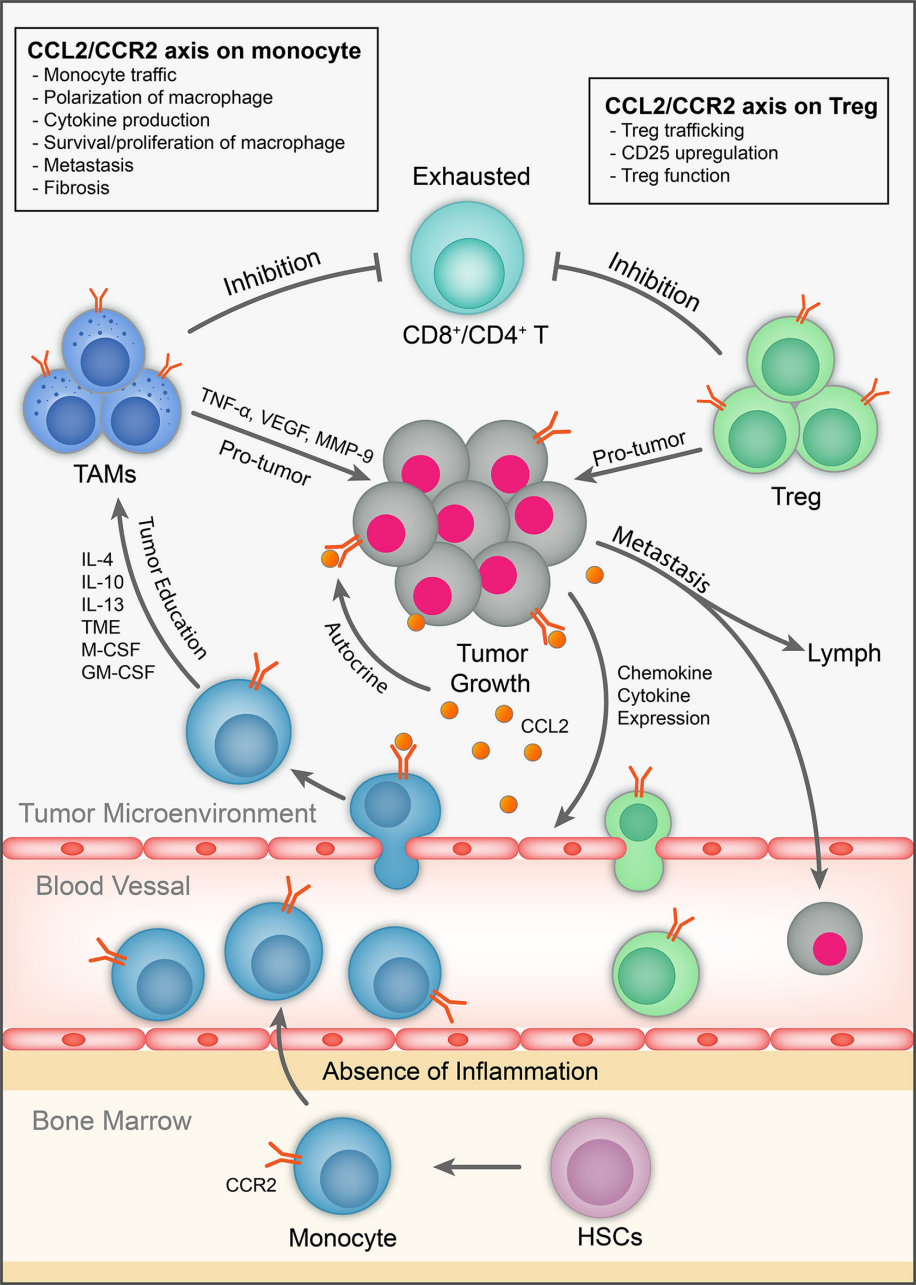
The role of CCL2/CCR2 axis in tumor immunology. CCL2 is expressed by immune cells, cancer and stromal cells in the TME. It exhibits chemotactic and non-chemotactic effects on CCR2-expressing monocytes/macrophages. It also has chemotactic and non-chemotactic effects on CCR2-expressing T cells, particularly Tregs. It also induces tumor cell proliferation/survival and metastasis in autocrine and paracrine fashion.

Despite the setbacks in development of CCL2/CCR2 in clinical studies, particularly for inflammatory conditions, the detrimental roles of the CCL2/CCR2 axis in cancer setting have increasingly been appreciated so that the CCL2/CCR2 axis remains to be actively targeted as candidates for immunotherapy (28) (**Tables 2**, **3**). We surmise above that CCR2 has enhancing effects on two main types of immunosuppressive immune cells to promote tumor progression and metastasis. Thus, together with direct effects on tumor cells, targeting the CCL2/CCR2 axis likely generates multiple attacks on multiple unfavorable cell types (including cancer cells) within the TME (**Figure 2**). In view of the rather disappointing developmental history of CCR2 antagonism, and the now increased understanding of mode of action of the CCL2/CCR2 axis, we attempt to discuss the strategies that could facilitate tailored application of CCR2 antagonism in cancer therapy.

### Can Elimination of CCR2-Expressing Cells Offer Advantage Over Functional Blocking of CCR2?

In a study dissecting the origin of TAM (84), the TAM percentage in CCR2-/- mice in the MMTV-PyMT (PyMT) mammary tumor model was found not to be significantly different compared to WT controls. However, in CCR2-DTR PyMT mice expressing diphtheria toxin receptor (DTR) under control of the Ccr2 locus, DT (diphtheria toxin) treatment resulted in a large depletion of tumor-associated monocytes and significant reduction of TAM numbers (84). CCR2 expression by TAMs could also render them sensitive to DT treatment (191). Thus, there is a difference between functional deficiency of CCR2 and elimination of CCR2^+^ myeloid cells (either TAM precursors or TAM themselves). Similarly, deletion of TAMs but not CCR2 deficiency slows the growth of mouse lung adenocarcinomas (192). Further, the same contrast of deletion vs inhibition could also apply to CCR2 expressing Tregs and tumor cells. We need to consider whether elimination of CCR2-expressing cells offers advantage over functional blocking when developing a CCR2 antagonist.

For clinical studies, how can we achieve the above different outcomes? Arguably, classical CCR2 antagonists could only block CCR2 function, but not directly delete the CCR2-expressing cells. Similarly, neutralization Abs to CCL2 are also unlikely deplete CCR2-expressing cells. On the other hand, administration of an anti-CCR2 antibody could drastically decrease the total number of monocytes/MØs in the peritoneal fluid but not the number of infiltrating granulocytes and lymphocytes, although it was not conclusive whether that reduction was caused by direct deletion (193). The same Ab was used subsequently to delete Ly6C^+^ monocytes during infection (194). Most depleting Abs, likely containing a human IgG1 Fc region could trigger Fc-dependent effector mechanisms including complement-dependent cytotoxicity (CDC), antibody-dependent cell cytotoxicity (ADCC), and phagocytosis (195). Thus, design of proper anti-CCR2 Abs needs taking mode of action into consideration. As several anti-CCR2 Abs are under development (**Table 3**), it would be of interest whether anti-tumor efficacy links to their cell depleting property, and whether the depleting effect of anti-CCR2 Abs could be enhanced by antibody drug conjugation *via* antibody-guided delivery of cytotoxic drugs. Of note, anti-human CD64 antibody (clone H22) conjugated to a cytotoxic agent dimeric pyrrolobenzodiazepine (dPBD) depleted CD163^+^ TAMs in humanized mice inoculated with human oral squamous cell carcinoma HSC-4 cells (196). Thus, the depleting effect of anti-CCR2 Abs could be enhanced by antibody drug conjugation *via* antibody-guided delivery of cytotoxic drugs.

Depletion of suppressive immune cells by targeting CCL2/CCR2 axis could also consider Treg cells. Within the TME, Treg cells consist of a large proportion of human tumor CD4^+^ infiltrates (197). Importantly, deletion of Tregs abrogates immunological unresponsiveness and promotes anti-tumor response (198). As discussed, Tregs express CCR2 (16, 118) and tumor Tregs contained an even higher proportion of CCR2^+^ Tregs (13). Importantly, CCR2 preferentially mediates tissue homing of Tregs including tumor tissue (13, 119, 120). Radiotherapy in cancer induces the recruitment of CCR2^+^ Tregs that contribute to treatment resistance (199). Notably, a phase 1 trial with a CCR2 antagonist in non-metastatic pancreatic cancer patients demonstrated that CCR2 inhibition decreases the number of tumor-infiltrating Tregs (200). Apart from Treg trafficking, CCR2 also regulates CD25 expression on Tregs and Treg function (119). Together, it may be important to consider the role of CCR2 in regulating traffic and function of Tregs when targeting CCR2 in cancer therapy.

### Does the Promiscuity of CCL2 and CCR2 Partnership Affect the Targeting Efficacy?

As discussed above, although CCL2 is a prototype CCR2 ligand, CCR2 can also partner with several other CCR2 agonists including CCL7 (MCP-3), CCL8 (MCP-2), CCL13 (MCP-4) and CCL12 (MCP-5) (201). Interestingly, deficiency of CCR2 ligand CCL7 but not CCL8 and CCL12 could reduce monocyte egress from bone marrow (87). Conversely, CCL2 has been reported to bind CCR4 on activated Tregs (202) and CD8^+^ T cells (203). Blocking the CCL2/CCR4 axis in Tregs evokes an anti-tumor immune response in a model of head and neck squamous cell carcinoma (202) while blocking of CCL2/CCR4 axis on CTLs could be detrimental as argued in the study (203). As a further complication, human CCR2 has two isoforms. As aforementioned, induction of CCR2A and CCR2B and signaling outcomes of CCR2A and CCR2B could be different. It remains to be known whether these underappreciated aspects of the mode of action of CCR2 and CCL2 could impact on the efficacy of different targeting strategies.

Related to the above context, significant and broader redundancy exists for cross talk between various chemokine receptors and ligands (10). Many chemokine receptors such as CCR1, CCR2, CCR5 and CXCR3 have all been implicated in the pathophysiology of multiple sclerosis and rheumatoid arthritis. It leads to the proposal to target more than one chemokine receptor (10). Similarly, the redundancy of the chemokines is also implicated. For example, as CCL7 acts a ligand for CCR2 for monocyte traffic (87), CCL7 can also act as a ligand for CCR1, CCR3, CCR5 and CCR10 (204). In a model of lung metastasis of breast cancer, CCR2^+^ MØs upon activation by CCL2 produce CCL3 to induce CCR1-mediated TAM retention in the lung (205), supporting involvement of multiple chemokines and receptors in tumor metastasis. Thus, combination therapies targeting multiple chemokines/chemokine receptors in the form of a single molecule had been proposed to counter such redundancy (10). Recent advances in engineering of bispecific, trispecific and even tetraspecific antibodies as a single molecule (206) may help to realize such a tenet.

### Does CCR2 Antagonism Preferentially Affect Primary Tumor and Metastasis

It has been shown that tumor-derived CCL2 supports tumor growth, depending on CCR2 expression by host cells (136) in mouse models of transferred liver cancer cells. Treatment with a CCR2 antagonist reduced the liver tumor volume, inhibited the abdominal dissemination and metastasis of cancer cells and reduced the recurrence of HCC after surgery (136). Interestingly, in the model, the CCR2 antagonist had no therapeutic effect on tumor cells with no CCL2 expression. Thus, the CCL2/CCR2 axis has effect on control of primary tumor. Nevertheless, an adoptive transfer study indicated that inflammatory monocytes preferentially migrate to the lung with metastatic tumors over the primary tumor (12). Further, CCL2 produced by MØs is required for metastatic seeding of cancer cells rather than tumor growth at the primary and metastatic sites (205). Similarly, in another study with breast cancer lines, CCL2 overexpression by tumor cells promoted cancer metastasis to lung and bone. Accordingly, CCL2 neutralization reduces metastasis (207). Thus, the CCL2/CCR2 axis has a critical role in the multiple stages of cancer metastasis (201).

Immunotherapy of cancer has also been explored with local administration of immunotherapies into the tumor (208). Local depletion of Treg cells inside the tumor led to the eradication of highly aggressive tumors and the development of persistent antitumor memory in a mouse model (209). Local depletion of MØs has been attempted to manipulate local immune responses in non-tumor models (210, 211). Conceivably, local depletion of CCR2^+^ TAM, Treg and tumor cells as well as local neutralization of ligand CCL2 in TME will likely enhance anti-tumor arm of immunity.

Although CCR2 antagonism in cancer is predominantly discussed in the context of solid tumors, the CCL2/CCR2 axis is also implicated in blood cancers. Not surprisingly, human monocytoid AML samples expressed CCR2 and CCL2 (212). Like solid tumors, the CCL2/CCR2 axis in AML also affects MØ phenotype in leukemia-bearing mice (213). Consequently, blockade of the CCL2/CCR2 axis affects migration and signaling of AML cells and MØs (213). In addition, CCR2 is expressed on a small fraction of human B lymphocytes in peripheral blood (PB) and tonsils (21, 214) and the cell-surface expression of CCR2 in different types of B-cell lymphomas can be rather high in patients with chronic lymphocytic leukemia (CLL) (215). Recruitment of monocytes towards the CLL lesion is also mediated by CCR2 (216). In a mouse model of multiple myeloma, osteoclasts have been found to express CCR2-targeting chemokines, and an anti-CCR2 monoclonal antibody was able to block osteoclast chemoattractant activity for multiple myeloma cells (61). Thus, there is evidence for the role of the CCL2/CCR2 axis in blood cancers.

### Can Combinational Therapy Broaden Application of CCR2 Antagonism in Cancer Therapy?

Development of CCR2 antagonism as monotherapy in autoimmune and inflammatory diseases has not been very successful, owing to many factors (10). As we highlighted above some aspects of how to target CCL2/CCR2 in cancer, we also contend that combination therapy may broaden and improve application of CCR2 antagonism as cancer therapy.

CCR2 antagonism has previously been combined with immune checkpoint inhibitors. In a mouse model transferred with bladder cancer line, combination therapy of a CCR2 antagonist and an anti-PD-L1 antibody showed significant synergy (217). The same combination also reduced murine melanoma pulmonary metastases and mammary fat pad tumor growth of implanted breast cancer cells (217). Notably, for bladder cancer growth and pulmonary metastases in the study, treatment with anti-PD-L1 did not overtly offer benefit (217). Similarly, in another study with glioma, tumor growth was not significantly inhibited by anti-PD-L1 monotherapy. However, anti-PD-L1 antibodies had a strong anti-tumor effect when combined with CCR2 deficiency or CCR2 inhibition (90). Immunologically, only combination treatment significantly enhanced intratumour T-cell responses (90). Synergy between CCR2 inhibition and immune checkpoint inhibitors was also reported for cutaneous T-cell lymphoma in a murine model (218). Collectively, these recent studies advocate targeting the CCL2/CCR2 axis to improve current immunotherapy with immune checkpoint inhibitors.

As combination with immune checkpoint inhibitors is a prime consideration, can CCR2 antagonism be combined with other strategies of immune modulation? Pancreatic ductal adenocarcinoma (PDAC) is considered to be resistant to immunotherapy. A recent study has highlighted the severe deficiency of conventional dendritic cells (cDC) with anti-tumor properties, specifically cDC1s in the presence of high numbers of suppressive TAMs in both murine model and human patient samples (219). Mobilizing cDCs into early pancreatic lesions with Flt3L administration can improve IFN-γ producing T cell response and disease stabilization. Furthermore, combination of a STING agonist (RR-S2-CDA) or CD40-agonist with FLt3L further increased the influx of cDC1s and enhanced intratumoural CD8^+^ CTL and CD4^+^ Th cell infiltration without Treg induction (219). Considering the positive outcome with CCR2 inhibition in pancreatic cancer (200), we speculate that a combination of CCR2 inhibition with the above strategies to increase cDCs would further warm up the TME in favor of anti-tumor responses.

Notably, changes in the TME could alter responsiveness to conventional cancer therapy. In the above study, cDC-directed therapy increased responsiveness of pancreatic cancer to radiation therapy (219). In the context of the CCL2/CCR2 axis, CCR2-/- mice have been found to respond better to treatment with doxorubicin or cisplatin (220). On the other hand, doxorubicin treatment can lead to CCL2 production by stromal cells from tumor microenvironment and contributes to drug resistance by recruitment of suppressive myeloid cells (220). These studies provide a rationale to combine CCR2 antagonism and chemotherapy. In clinical trials in pancreatic patients with a CCR2 inhibitor, combination therapy with FOLFIRINOX showed better overall survival compared to FOLFIRINOX monotherapy (200). Notably, clinical trials with anti-CCL2 antibody Carlumab, with or without combination chemotherapy did not offer significant efficacy, probably due to lack of long-term neutralization of CCL2 (221) or redundancy issue discussed above. Similar to chemotherapy, Radiotherapy could induce the CCR2-dependent recruitment of monocytes and CCR2^+^ Tregs into the tumor (199). Similarly, radiotherapy increased the release of chemokines CCL2 and CCL5 in experimental tumor models. Fittingly, blockade of CCR2 and CCR5 improved the efficacy of radiotherapy in radioresponsive tumors (222). Together, evidence supports combining CCR2 antagonism with chemotherapy and radiotherapy.

## Overview/Conclusions

CCR2 antagonism has been investigated as a therapeutic for autoimmune diseases and more recently for cancer over many decades. Suffice to say, clinical trials so far have not been very successful (10, 189, 223, 224). There are many factors contributing to the results (10). In the context of cancer, we highlight here the complex and sometimes underappreciated roles of the CCL2/CCR2 axis in regulating abundance and functions of monocytes/MØs, T cells (particularly Tregs) and tumor cells (**Figure 2**). With the increase in the understanding of modes of action, expression regulation of CCL2/CCR2 in various immune cells, other stromal cells, and cancer cells, and improved and versatile delivery platforms including multiple specific Ab as a single biologic, antibody drug conjugation and PROTAC, as well as identification of biomarkers with predictive value, we contend that there are still potential solutions to generating effective therapeutics targeting CCR2/CCL2 axis in cancer.

## Author Contributions

LF, XR, HY, and YZ wrote the manuscript. XR made the figures. XR, HY, and YZ made the tables. YZ supervised the writing. All authors contributed to the article and approved the submitted version.

## Conflict of Interest

All authors were employed by Shanghai Huaota Biopharm during the preparation of this article.

## Publisher’s Note

All claims expressed in this article are solely those of the authors and do not necessarily represent those of their affiliated organizations, or those of the publisher, the editors and the reviewers. Any product that may be evaluated in this article, or claim that may be made by its manufacturer, is not guaranteed or endorsed by the publisher.

## References

[1] Ozga AJ Chow MT Luster AD. Chemokines and the Immune Response to Cancer. Immunity (2021) 54(5):859–74. doi: 10.1016/j.immuni.2021.01.012 PMC8434759 33838745

[2] Shi C Pamer EG. Monocyte Recruitment During Infection and Inflammation. Nat Rev Immunol (2011) 11(11):762–74. doi: 10.1038/nri3070 PMC3947780 21984070

[3] Kurihara T Warr G Loy J Bravo R. Defects in Macrophage Recruitment and Host Defense in Mice Lacking the CCR2 Chemokine Receptor. J Exp Med (1997) 186(10):1757–62. doi: 10.1084/jem.186.10.1757 PMC2199145 9362535

[4] Kuziel WA Morgan SJ Dawson TC Griffin S Smithies O Ley K. Severe Reduction in Leukocyte Adhesion and Monocyte Extravasation in Mice Deficient in CC Chemokine Receptor 2. Proc Natl Acad Sci USA (1997) 94(22):12053–8. doi: 10.1073/pnas.94.22.12053 PMC23699 9342361

[5] Si Y Tsou CL Croft K Charo IF. CCR2 Mediates Hematopoietic Stem and Progenitor Cell Trafficking to Sites of Inflammation in Mice. J Clin Invest (2010) 120(4):1192–203. doi: 10.1172/JCI40310 PMC2846049 20234092

[6] Boring L Gosling J Chensue SW Kunkel SL Farese RV Jr. Broxmeyer HE. Impaired Monocyte Migration and Reduced Type 1 (Th1) Cytokine Responses in C-C Chemokine Receptor 2 Knockout Mice. J Clin Invest (1997) 100(10):2552–61. doi: 10.1172/JCI119798 PMC508456 9366570

[7] Izikson L Klein RS Charo IF Weiner HL Luster AD. Resistance to Experimental Autoimmune Encephalomyelitis in Mice Lacking the CC Chemokine Receptor (CCR)2. J Exp Med (2000) 192(7):1075–80. doi: 10.1084/jem.192.7.1075 PMC2193310 11015448

[8] Hohl TM Rivera A Lipuma L Gallegos A Shi C Mack M. Inflammatory Monocytes Facilitate Adaptive CD4 T Cell Responses During Respiratory Fungal Infection. Cell Host Microbe (2009) 6(5):470–81. doi: 10.1016/j.chom.2009.10.007 PMC2785497 19917501

[9] Ko HJ Brady JL Ryg-Cornejo V Hansen DS Vremec D Shortman K. GM-CSF-Responsive Monocyte-Derived Dendritic Cells Are Pivotal in Th17 Pathogenesis. J Immunol (2014) 192(5):2202–9. doi: 10.4049/jimmunol.1302040 24489100

[10] Horuk R. Chemokine Receptor Antagonists: Overcoming Developmental Hurdles. Nat Rev Drug Discov (2009) 8(1):23–33. doi: 10.1038/nrd2734 19079127

[11] Quinones MP Estrada CA Kalkonde Y Ahuja SK Kuziel WA Mack M. The Complex Role of the Chemokine Receptor CCR2 in Collagen-Induced Arthritis: Implications for Therapeutic Targeting of CCR2 in Rheumatoid Arthritis. J Mol Med (2005) 83(9):672–81. doi: 10.1007/s00109-005-0637-5 15827759

[12] Qian B-Z Li J Zhang H Kitamura T Zhang J Campion LR. CCL2 Recruits Inflammatory Monocytes to Facilitate Breast-Tumor Metastasis. Nature (2011) 475(7355):222–5. doi: 10.1038/nature10138 PMC3208506 21654748

[13] Loyher P-L Rochefort J Baudesson de Chanville C Hamon P Lescaille G Bertolus C. CCR2 Influences T Regulatory Cell Migration to Tumors and Serves as a Biomarker of Cyclophosphamide Sensitivity. Cancer Res (2016) 76(22):6483–94. doi: 10.1158/0008-5472.CAN-16-0984 27680685

[14] Charo IF Myers SJ Herman A Franci C Connolly AJ Coughlin SR. Molecular Cloning and Functional Expression of Two Monocyte Chemoattractant Protein 1 Receptors Reveals Alternative Splicing of the Carboxyl-Terminal Tails. Proc Natl Acad Sci (1994) 91(7):2752–6. doi: 10.1073/pnas.91.7.2752 PMC43448 8146186

[15] Yamasaki R Liu L Lin J Ransohoff RM. Role of CCR2 in Immunobiology and Neurobiology. Clin Exp Neuroimmunol (2012) 3(1):16–29. doi: 10.1111/j.1759-1961.2011.00024.x

[16] Lee JH Kang SG Kim CH. FoxP3+ T Cells Undergo Conventional First Switch to Lymphoid Tissue Homing Receptors in Thymus But Accelerated Second Switch to Nonlymphoid Tissue Homing Receptors in Secondary Lymphoid Tissues. J Immunol (2007) 178(1):301–11. doi: 10.4049/jimmunol.178.1.301 17182567

[17] Bonecchi R Bianchi G Bordignon PP D'Ambrosio D Lang R Borsatti A. Differential Expression of Chemokine Receptors and Chemotactic Responsiveness of Type 1 T Helper Cells (Th1s) and Th2s. J Exp Med (1998) 187(1):129–34. doi: 10.1084/jem.187.1.129 PMC2199181 9419219

[18] Nansen A Marker O Bartholdy C Thomsen AR. CCR2+ and CCR5+ CD8+ T Cells Increase During Viral Infection and Migrate to Sites of Infection. Eur J Immunol (2000) 30(7):1797–806. doi: 10.1002/1521-4141(200007)30:7<1797::AID-IMMU1797>3.0.CO;2-B 10940868

[19] Metelitsa LS Wu HW Wang H Yang Y Warsi Z Asgharzadeh S. Natural Killer T Cells Infiltrate Neuroblastomas Expressing the Chemokine CCL2. J Exp Med (2004) 199(9):1213–21. doi: 10.1084/jem.20031462 PMC2211904 15123743

[20] McKenzie DR Kara EE Bastow CR Tyllis TS Fenix KA Gregor CE. IL-17-Producing γδ T Cells Switch Migratory Patterns Between Resting and Activated States. Nat Commun (2017) 8:15632. doi: 10.1038/ncomms15632 28580944 PMC5465362

[21] Frade J Mellado M del Real G Gutierrez-Ramos J Lind P Martinez-A C. Characterization of the CCR2 Chemokine Receptor: Functional CCR2 Receptor Expression in B Cells. J Immunol (1997) 159(11):5576–84. 9548499

[22] Swiecki M Miller HL Sesti-Costa R Cella M Gilfillan S Colonna M. Microbiota Induces Tonic CCL2 Systemic Levels That Control pDC Trafficking in Steady State. Mucosal Immunol (2017) 10(4):936–45. doi: 10.1038/mi.2016.99 PMC5423869 27827374

[23] Pan Q Feng Y Peng Y Zhou H Deng Z Li L. Basophil Recruitment to Skin Lesions of Patients With Systemic Lupus Erythematosus Mediated by CCR1 and CCR2. Cell Physiol Biochem (2017) 43(2):832–9. doi: 10.1159/000481609 28954264

[24] Belema-Bedada F Uchida S Martire A Kostin S Braun T. Efficient Homing of Multipotent Adult Mesenchymal Stem Cells Depends on FROUNT-Mediated Clustering of CCR2. Cell Stem Cell (2008) 2(6):566–75. doi: 10.1016/j.stem.2008.03.003 18522849

[25] Weber KS Nelson PJ Gröne H-J Weber C. Expression of CCR2 by Endothelial Cells: Implications for MCP-1 Mediated Wound Injury Repair and *In Vivo* Inflammatory Activation of Endothelium. Arteriosclerosis thrombosis Vasc Biol (1999) 19(9):2085–93. doi: 10.1161/01.ATV.19.9.2085 10479649

[26] El Khoury J Toft M Hickman SE Means TK Terada K Geula C. Ccr2 Deficiency Impairs Microglial Accumulation and Accelerates Progression of Alzheimer-Like Disease. Nat Med (2007) 13(4):432–8. doi: 10.1038/nm1555 17351623

[27] Warren GL Hulderman T Mishra D Gao X Millecchia L O'Farrell L. Chemokine Receptor CCR2 Involvement in Skeletal Muscle Regeneration. FASEB J (2005) 19(3):413–5. doi: 10.1096/fj.04-2421fje 15601671

[28] Hao Q Vadgama JV Wang P. CCL2/CCR2 Signaling in Cancer Pathogenesis. Cell Commun Signal (2020) 18(1):82. doi: 10.1186/s12964-020-00589-8 32471499 PMC7257158

[29] Chen Y Green SR Ho J Li A Almazan F Quehenberger O. The Mouse CCR2 Gene Is Regulated by Two Promoters That Are Responsive to Plasma Cholesterol and Peroxisome Proliferator-Activated Receptor Gamma Ligands. Biochem Biophys Res Commun (2005) 332(1):188–93. doi: 10.1016/j.bbrc.2005.04.110 15896316

[30] Wenstedt EF Verberk SG Kroon J Neele AE Baardman J Claessen N. Salt Increases Monocyte CCR2 Expression and Inflammatory Responses in Humans. JCI Insight (2019) 4(21). doi: 10.1172/jci.insight.130508 PMC6948772 31672939

[31] Araya P Romero J Delgado-López F Gonzalez I Añazco C Perez R. HMGB1 Decreases CCR-2 Expression and Migration of M2 Macrophages Under Hypoxia. Inflamm Res (2019) 68(8):639–42. doi: 10.1007/s00011-019-01249-5 31115587

[32] Sozzani S Luini W Borsatti A Polentarutti N Zhou D Piemonti L. Receptor Expression and Responsiveness of Human Dendritic Cells to a Defined Set of CC and CXC Chemokines. J Immunol (1997) 159(4):1993–2000. 9257866

[33] Franci C Wong LM Van Damme J Proost P Charo IF. Monocyte Chemoattractant Protein-3, But Not Monocyte Chemoattractant Protein-2, Is a Functional Ligand of the Human Monocyte Chemoattractant Protein-1 Receptor. J Immunol (1995) 154(12):6511–7. 7759884

[34] Combadiere C Ahuja SK Van Damme J Tiffany HL Gao JL Murphy PM. Monocyte Chemoattractant Protein-3 Is a Functional Ligand for CC Chemokine Receptors 1 and 2B. J Biol Chem (1995) 270(50):29671–5. doi: 10.1074/jbc.270.50.29671 8530354

[35] Gong X Gong W Kuhns DB Ben-Baruch A Howard OM Wang JM. Monocyte Chemotactic Protein-2 (MCP-2) Uses CCR1 and CCR2B as Its Functional Receptors. J Biol Chem (1997) 272(18):11682–5. doi: 10.1074/jbc.272.18.11682 9115216

[36] Berkhout TA Sarau HM Moores K White JR Elshourbagy N Appelbaum E. Cloning, *In Vitro* Expression, and Functional Characterization of a Novel Human CC Chemokine of the Monocyte Chemotactic Protein (MCP) Family (MCP-4) That Binds and Signals Through the CC Chemokine Receptor 2B. J Biol Chem (1997) 272(26):16404–13. doi: 10.1074/jbc.272.26.16404 9195948

[37] Nomiyama H Hieshima K Nakayama T Sakaguchi T Fujisawa R Tanase S. Human CC Chemokine Liver-Expressed Chemokine/CCL16 Is a Functional Ligand for CCR1, CCR2 and CCR5, and Constitutively Expressed by Hepatocytes. Int Immunol (2001) 13(8):1021–9. doi: 10.1093/intimm/13.8.1021 11470772

[38] Dommel S Blüher M. Does C-C Motif Chemokine Ligand 2 (CCL2) Link Obesity to a Pro-Inflammatory State? Int J Mol Sci (2021) 22(3):1500. doi: 10.3390/ijms22031500 33540898 PMC7867366

[39] Reiter E Lefkowitz RJ. GRKs and Beta-Arrestins: Roles in Receptor Silencing, Trafficking and Signaling. Trends Endocrinol Metab (2006) 17(4):159–65. doi: 10.1016/j.tem.2006.03.008 16595179

[40] O'Boyle G Brain JG Kirby JA Ali S. Chemokine-Mediated Inflammation: Identification of a Possible Regulatory Role for CCR2. Mol Immunol (2007) 44(8):1944–53. doi: 10.1016/j.molimm.2006.09.033 17081610

[41] Thelen M. Dancing to the Tune of Chemokines. Nat Immunol (2001) 2(2):129–34. doi: 10.1038/84224 11175805

[42] Roca H Varsos Z Pienta KJ. CCL2 Protects Prostate Cancer PC3 Cells From Autophagic Death *via* Phosphatidylinositol 3-Kinase/AKT-Dependent Survivin Up-Regulation. J Biol Chem (2008) 283(36):25057–73. doi: 10.1074/jbc.M801073200 PMC2529129 18611860

[43] Loberg RD Day LL Harwood J Ying C John LNS Giles R. CCL2 Is a Potent Regulator of Prostate Cancer Cell Migration and Proliferation. Neoplasia (2006) 8(7):578–86. doi: 10.1593/neo.06280 PMC1601934 16867220

[44] Lin TH Liu HH Tsai TH Chen CC Hsieh TF Lee SS. CCL2 Increases Alphavbeta3 Integrin Expression and Subsequently Promotes Prostate Cancer Migration. Biochim Biophys Acta (2013) 1830(10):4917–27. doi: 10.1016/j.bbagen.2013.06.033 23845726

[45] Song G Ouyang G Bao S. The Activation of Akt/PKB Signaling Pathway and Cell Survival. J Cell Mol Med (2005) 9(1):59–71. doi: 10.1111/j.1582-4934.2005.tb00337.x 15784165 PMC6741304

[46] Sauvonnet N Lambermont I van der Bruggen P Cornelis GR. YopH Prevents Monocyte Chemoattractant Protein 1 Expression in Macrophages and T-Cell Proliferation Through Inactivation of the Phosphatidylinositol 3-Kinase Pathway. Mol Microbiol (2002) 45(3):805–15. doi: 10.1046/j.1365-2958.2002.03053.x 12139625

[47] Marino M Acconcia F Trentalance A. Biphasic Estradiol-Induced AKT Phosphorylation Is Modulated by PTEN *via* MAP Kinase in HepG2 Cells. Mol Biol Cell (2003) 14(6):2583–91. doi: 10.1091/mbc.e02-09-0621 PMC194905 12808053

[48] Kroemer G Levine B. Autophagic Cell Death: The Story of a Misnomer. Nat Rev Mol Cell Biol (2008) 9(12):1004–10. doi: 10.1038/nrm2529 PMC2727358 18971948

[49] Dasgupta P Kinkade R Joshi B Decook C Haura E Chellappan S. Nicotine Inhibits Apoptosis Induced by Chemotherapeutic Drugs by Up-Regulating XIAP and Survivin. Proc Natl Acad Sci USA (2006) 103(16):6332–7. doi: 10.1073/pnas.0509313103 PMC1458878 16601104

[50] Tamm I Wang Y Sausville E Scudiero DA Vigna N Oltersdorf T. IAP-Family Protein Survivin Inhibits Caspase Activity and Apoptosis Induced by Fas (CD95), Bax, Caspases, and Anticancer Drugs. Cancer Res (1998) 58(23):5315–20. 9850056

[51] Altieri DC. Survivin, Cancer Networks and Pathway-Directed Drug Discovery. Nat Rev Cancer (2008) 8(1):61–70. doi: 10.1038/nrc2293 18075512

[52] Fornaro M Plescia J Chheang S Tallini G Zhu YM King M. Fibronectin Protects Prostate Cancer Cells From Tumor Necrosis Factor-Alpha-Induced Apoptosis *via* the AKT/survivin Pathway. J Biol Chem (2003) 278(50):50402–11. doi: 10.1074/jbc.M307627200 14523021

[53] Chiu YC Shieh DC Tong KM Chen CP Huang KC Chen PC. Involvement of AdipoR Receptor in Adiponectin-Induced Motility and Alpha2beta1 Integrin Upregulation in Human Chondrosarcoma Cells. Carcinogenesis (2009) 30(10):1651–9. doi: 10.1093/carcin/bgp156 19549705

[54] Madrid LV Mayo MW Reuther JY Baldwin AS Jr. Akt Stimulates the Transactivation Potential of the RelA/p65 Subunit of NF-Kappa B Through Utilization of the Ikappa B Kinase and Activation of the Mitogen-Activated Protein Kinase P38. J Biol Chem (2001) 276(22):18934–40. doi: 10.1074/jbc.M101103200 11259436

[55] Tang CH Tsai CC. CCL2 Increases MMP-9 Expression and Cell Motility in Human Chondrosarcoma Cells *via* the Ras/Raf/MEK/ERK/NF-κb Signaling Pathway. Biochem Pharmacol (2012) 83(3):335–44. doi: 10.1016/j.bcp.2011.11.013 22138288

[56] Deryugina EI Quigley JP. Matrix Metalloproteinases and Tumor Metastasis. Cancer Metastasis Rev (2006) 25(1):9–34. doi: 10.1007/s10555-006-7886-9 16680569

[57] Friday BB Adjei AA. Advances in Targeting the Ras/Raf/MEK/Erk Mitogen-Activated Protein Kinase Cascade With MEK Inhibitors for Cancer Therapy. Clin Cancer Res (2008) 14(2):342–6. doi: 10.1158/1078-0432.CCR-07-4790 18223206

[58] Lee HW Ahn DH Crawley SC Li JD Gum JR Jr Basbaum CB. Phorbol 12-Myristate 13-Acetate Up-Regulates the Transcription of MUC2 Intestinal Mucin *via* Ras, ERK, and NF-Kappa B. J Biol Chem (2002) 277(36):32624–31. doi: 10.1074/jbc.M200353200 12077118

[59] Kunnumakkara AB Anand P Aggarwal BB. Curcumin Inhibits Proliferation, Invasion, Angiogenesis and Metastasis of Different Cancers Through Interaction With Multiple Cell Signaling Proteins. Cancer Lett (2008) 269(2):199–225. doi: 10.1016/j.canlet.2008.03.009 18479807

[60] Lee CY Huang CY Chen MY Lin CY Hsu HC Tang CH. IL-8 Increases Integrin Expression and Cell Motility in Human Chondrosarcoma Cells. J Cell Biochem (2011) 112(9):2549–57. doi: 10.1002/jcb.23179 21590707

[61] Mellado M Rodríguez-Frade JM Aragay A del Real G Martín AM Vila-Coro AJ. The Chemokine Monocyte Chemotactic Protein 1 Triggers Janus Kinase 2 Activation and Tyrosine Phosphorylation of the CCR2B Receptor. J Immunol (1998) 161(2):805–13. 9670957

[62] Agrawal S Gollapudi S Su H Gupta S. Leptin Activates Human B Cells to Secrete TNF-Alpha, IL-6, and IL-10 *via* JAK2/STAT3 and P38mapk/ERK1/2 Signaling Pathway. J Clin Immunol (2011) 31(3):472–8. doi: 10.1007/s10875-010-9507-1 PMC3132280 21243519

[63] Sanz-Moreno V Gaggioli C Yeo M Albrengues J Wallberg F Viros A. ROCK and JAK1 Signaling Cooperate to Control Actomyosin Contractility in Tumor Cells and Stroma. Cancer Cell (2011) 20(2):229–45. doi: 10.1016/j.ccr.2011.06.018 21840487

[64] Wolf MJ Hoos A Bauer J Boettcher S Knust M Weber A. Endothelial CCR2 Signaling Induced by Colon Carcinoma Cells Enables Extravasation *via* the JAK2-Stat5 and P38mapk Pathway. Cancer Cell (2012) 22(1):91–105. doi: 10.1016/j.ccr.2012.05.023 22789541

[65] Wong L-M Myers SJ Tsou C-L Gosling J Arai H Charo IF. Organization and Differential Expression of the Human Monocyte Chemoattractant Protein 1 Receptor Gene: Evidence For The Role Of The Carboxyl-terminal Tail In Receptor Trafficking*. J Biol Chem (1997) 272(2):1038–45. doi: 10.1074/jbc.272.2.1038 8995400

[66] Bartoli C Civatte M Pellissier JF Figarella-Branger D. CCR2A and CCR2B, the Two Isoforms of the Monocyte Chemoattractant Protein-1 Receptor Are Up-Regulated and Expressed by Different Cell Subsets in Idiopathic Inflammatory Myopathies. Acta Neuropathol (2001) 102(4):385–92. doi: 10.1007/s004010100394 11603815

[67] Cho ML Yoon BY Ju JH Jung YO Jhun JY Park MK. Expression of CCR2A, an Isoform of MCP-1 Receptor, Is Increased by MCP-1, CD40 Ligand and TGF-Beta in Fibroblast Like Synoviocytes of Patients With RA. Exp Mol Med (2007) 39(4):499–507. doi: 10.1038/emm.2007.55 17934338

[68] Liang Y Bollen AW Gupta N. CC Chemokine Receptor-2A Is Frequently Overexpressed in Glioblastoma. J Neuro-Oncol (2008) 86(2):153–63. doi: 10.1007/s11060-007-9463-7 17703277

[69] Sanders SK Crean SM Boxer PA Kellner D LaRosa GJ Hunt SW 3rd. Functional Differences Between Monocyte Chemotactic Protein-1 Receptor A and Monocyte Chemotactic Protein-1 Receptor B Expressed in a Jurkat T Cell. J Immunol (2000) 165(9):4877–83. doi: 10.4049/jimmunol.165.9.4877 11046012

[70] Murray Peter J Allen Judith E Biswas Subhra K Fisher Edward A Gilroy Derek W Goerdt S. Macrophage Activation and Polarization: Nomenclature and Experimental Guidelines. Immunity (2014) 41(1):14–20. doi: 10.1016/j.immuni.2014.06.008 25035950 PMC4123412

[71] Fleetwood AJ Lawrence T Hamilton JA Cook AD. Granulocyte-Macrophage Colony-Stimulating Factor (CSF) and Macrophage CSF-Dependent Macrophage Phenotypes Display Differences in Cytokine Profiles and Transcription Factor Activities: Implications for CSF Blockade in Inflammation. J Immunol (2007) 178(8):5245–52. doi: 10.4049/jimmunol.178.8.5245 17404308

[72] Menezes S Melandri D Anselmi G Perchet T Loschko J Dubrot J. The Heterogeneity of Ly6C(hi) Monocytes Controls Their Differentiation Into iNOS(+) Macrophages or Monocyte-Derived Dendritic Cells. Immunity (2016) 45(6):1205–18. doi: 10.1016/j.immuni.2016.12.001 PMC5196026 28002729

[73] Verreck FA de Boer T Langenberg DM Hoeve MA Kramer M Vaisberg E. Human IL-23-Producing Type 1 Macrophages Promote But IL-10-Producing Type 2 Macrophages Subvert Immunity to (Myco) Bacteria. Proc Natl Acad Sci (2004) 101(13):4560–5. doi: 10.1073/pnas.0400983101 PMC384786 15070757

[74] Croxford AL Lanzinger M Hartmann FJ Schreiner B Mair F Pelczar P. The Cytokine GM-CSF Drives the Inflammatory Signature of CCR2+ Monocytes and Licenses Autoimmunity. Immunity (2015) 43(3):502–14. doi: 10.1016/j.immuni.2015.08.010 26341401

[75] Martinez FO Gordon S. The M1 and M2 Paradigm of Macrophage Activation: Time for Reassessment. F1000prime Rep (2014) 6. doi: 10.12703/P6-13 PMC3944738 24669294

[76] Bayne LJ Beatty GL Jhala N Clark CE Rhim AD Stanger BZ. Tumor-Derived Granulocyte-Macrophage Colony-Stimulating Factor Regulates Myeloid Inflammation and T Cell Immunity in Pancreatic Cancer. Cancer Cell (2012) 21(6):822–35. doi: 10.1016/j.ccr.2012.04.025 PMC3575028 22698406

[77] Bronte V Chappell DB Apolloni E Cabrelle A Wang M Hwu P. Unopposed Production of Granulocyte-Macrophage Colony-Stimulating Factor by Tumors Inhibits CD8+ T Cell Responses by Dysregulating Antigen-Presenting Cell Maturation. J Immunol (1999) 162(10):5728–37. PMC2228333 10229805

[78] Braun MC Lahey E Kelsall BL. Selective Suppression of IL-12 Production by Chemoattractants. J Immunol (2000) 164(6):3009–17. doi: 10.4049/jimmunol.164.6.3009 10706689

[79] Bronte V Brandau S Chen SH Colombo MP Frey AB Greten TF. Recommendations for Myeloid-Derived Suppressor Cell Nomenclature and Characterization Standards. Nat Commun (2016) 7:12150. doi: 10.1038/ncomms12150 27381735 PMC4935811

[80] Tcyganov E Mastio J Chen E Gabrilovich DI. Plasticity of Myeloid-Derived Suppressor Cells in Cancer. Curr Opin Immunol (2018) 51:76–82. doi: 10.1016/j.coi.2018.03.009 29547768 PMC5943174

[81] Cortez-Retamozo V Etzrodt M Newton A Rauch PJ Chudnovskiy A Berger C. Origins of Tumor-Associated Macrophages and Neutrophils. Proc Natl Acad Sci (2012) 109(7):2491–6. doi: 10.1073/pnas.1113744109 PMC3289379 22308361

[82] Movahedi K Laoui D Gysemans C Baeten M Stangé G Van den Bossche J. Different Tumor Microenvironments Contain Functionally Distinct Subsets of Macrophages Derived From Ly6C (High) Monocytes. Cancer Res (2010) 70(14):5728–39. doi: 10.1158/0008-5472.CAN-09-4672 20570887

[83] Zhou W Ke SQ Huang Z Flavahan W Fang X Paul J. Periostin Secreted by Glioblastoma Stem Cells Recruits M2 Tumor-Associated Macrophages and Promotes Malignant Growth. Nat Cell Biol (2015) 17(2):170–82. doi: 10.1038/ncb3090 PMC4312504 25580734

[84] Franklin RA Liao W Sarkar A Kim MV Bivona MR Liu K. The Cellular and Molecular Origin of Tumor-Associated Macrophages. Science (2014) 344(6186):921–5. doi: 10.1126/science.1252510 PMC4204732 24812208

[85] Zhu Y Herndon JM Sojka DK Kim KW Knolhoff BL Zuo C. Tissue-Resident Macrophages in Pancreatic Ductal Adenocarcinoma Originate From Embryonic Hematopoiesis and Promote Tumor Progression. Immunity (2017) 47(2):323–38.e6. doi: 10.1016/j.immuni.2017.07.014 28813661 PMC5578409

[86] Lin Y Xu J Lan H. Tumor-Associated Macrophages in Tumor Metastasis: Biological Roles and Clinical Therapeutic Applications. J Hematol Oncol (2019) 12(1):76. doi: 10.1186/s13045-019-0760-3 31300030 PMC6626377

[87] Tsou C-L Peters W Si Y Slaymaker S Aslanian AM Weisberg SP. Critical Roles for CCR2 and MCP-3 in Monocyte Mobilization From Bone Marrow and Recruitment to Inflammatory Sites. J Clin Invest (2007) 117(4):902–9. doi: 10.1172/JCI29919 PMC1810572 17364026

[88] Lu B Rutledge BJ Gu L Fiorillo J Lukacs NW Kunkel SL. Abnormalities in Monocyte Recruitment and Cytokine Expression in Monocyte Chemoattractant Protein 1–Deficient Mice. J Exp Med (1998) 187(4):601–8. doi: 10.1084/jem.187.4.601 PMC2212142 9463410

[89] Shand FH Ueha S Otsuji M Koid SS Shichino S Tsukui T. Tracking of Intertissue Migration Reveals the Origins of Tumor-Infiltrating Monocytes. Proc Natl Acad Sci USA (2014) 111(21):7771–6. doi: 10.1073/pnas.1402914111 PMC4040600 24825888

[90] Flores-Toro JA Luo D Gopinath A Sarkisian MR Campbell JJ Charo IF. CCR2 Inhibition Reduces Tumor Myeloid Cells and Unmasks a Checkpoint Inhibitor Effect to Slow Progression of Resistant Murine Gliomas. Proc Natl Acad Sci (2020) 117(2):1129–38. doi: 10.1073/pnas.1910856117 PMC6969504 31879345

[91] Ueno T Toi M Saji H Muta M Bando H Kuroi K. Significance of Macrophage Chemoattractant Protein-1 in Macrophage Recruitment, Angiogenesis, and Survival in Human Breast Cancer. Clin Cancer Res (2000) 6(8):3282–9. 10955814

[92] Saji H Koike M Yamori T Saji S Seiki M Matsushima K. Significant Correlation of Monocyte Chemoattractant Protein-1 Expression With Neovascularization and Progression of Breast Carcinoma. Cancer: Interdiscip Int J Am Cancer Soc (2001) 92(5):1085–91. doi: 10.1002/1097-0142(20010901)92:5<1085::AID-CNCR1424>3.0.CO;2-K 11571719

[93] Valković T Fučkar D Štifter S Matušan K Hasan M Dobrila F. Macrophage Level Is Not Affected by Monocyte Chemotactic Protein-1 in Invasive Ductal Breast Carcinoma. J Cancer Res Clin Oncol (2005) 131(7):453–8. doi: 10.1007/s00432-004-0667-3 PMC12161258 15883814

[94] Yang H Zhang Q Xu M Wang L Chen X Feng Y. CCL2-CCR2 Axis Recruits Tumor Associated Macrophages to Induce Immune Evasion Through PD-1 Signaling in Esophageal Carcinogenesis. Mol Cancer (2020) 19(1):41. doi: 10.1186/s12943-020-01165-x 32103760 PMC7045401

[95] Gschwandtner M Derler R Midwood KS. More Than Just Attractive: How CCL2 Influences Myeloid Cell Behavior Beyond Chemotaxis. Front Immunol (2019) 10:2759. doi: 10.3389/fimmu.2019.02759 PMC6923224 31921102

[96] Patel AA Zhang Y Fullerton JN Boelen L Rongvaux A Maini AA. The Fate and Lifespan of Human Monocyte Subsets in Steady State and Systemic Inflammation. J Exp Med (2017) 214(7):1913–23. doi: 10.1084/jem.20170355 PMC5502436 28606987

[97] Yona S Kim K-W Wolf Y Mildner A Varol D Breker M. Fate Mapping Reveals Origins and Dynamics of Monocytes and Tissue Macrophages Under Homeostasis. Immunity (2013) 38(1):79–91. doi: 10.1016/j.immuni.2012.12.001 23273845 PMC3908543

[98] Carrington EM Tarlinton DM Gray DH Huntington ND Zhan Y Lew AM. The Life and Death of Immune Cell Types: The Role of BCL-2 Anti-Apoptotic Molecules. Immunol Cell Biol (2017) 95(10):870–7. doi: 10.1038/icb.2017.72 28875977

[99] Thorp E Li Y Bao L Yao PM Kuriakose G Rong J. Brief Report: Increased Apoptosis in Advanced Atherosclerotic Lesions of Apoe–/– Mice Lacking Macrophage Bcl-2. Arteriosclerosis thrombosis Vasc Biol (2009) 29(2):169–72. doi: 10.1161/ATVBAHA.108.176495 PMC2731712 18988889

[100] Shearn AI Deswaerte V Gautier EL Saint-Charles F Pirault J Bouchareychas L. Bcl-X Inactivation in Macrophages Accelerates Progression of Advanced Atherosclerotic Lesions in Apoe(-/-) Mice. Arterioscler Thromb Vasc Biol (2012) 32(5):1142–9. doi: 10.1161/ATVBAHA.111.239111 22383704

[101] Speir M Lawlor KE Glaser SP Abraham G Chow S Vogrin A. Eliminating Legionella by Inhibiting BCL-XL to Induce Macrophage Apoptosis. Nat Microbiol (2016) 1:15034. doi: 10.1038/nmicrobiol.2015.34 27572165

[102] Steimer DA Boyd K Takeuchi O Fisher JK Zambetti GP Opferman JT. Selective Roles for Antiapoptotic MCL-1 During Granulocyte Development and Macrophage Effector Function. Blood J Am Soc Hematol (2009) 113(12):2805–15. doi: 10.1182/blood-2008-05-159145 PMC2661864 19064728

[103] Dzhagalov I St. John A He Y-W. The Antiapoptotic Protein Mcl-1 Is Essential for the Survival of Neutrophils But Not Macrophages. Blood (2007) 109(4):1620–6. doi: 10.1182/blood-2006-03-013771 PMC1794052 17062731

[104] Schenk RL Tuzlak S Carrington EM Zhan Y Heinzel S Teh CE. Characterisation of Mice Lacking All Functional Isoforms of the Pro-Survival BCL-2 Family Member A1 Reveals Minor Defects in the Haematopoietic Compartment. Cell Death Differ (2017) 24(3):534–45. doi: 10.1038/cdd.2016.156 PMC5344213 28085150

[105] Roca H Varsos ZS Sud S Craig MJ Ying C Pienta KJ. CCL2 and Interleukin-6 Promote Survival of Human CD11b+ Peripheral Blood Mononuclear Cells and Induce M2-Type Macrophage Polarization. J Biol Chem (2009) 284(49):34342–54. doi: 10.1074/jbc.M109.042671 PMC2797202 19833726

[106] Choi S You S Kim D Choi SY Kwon HM Kim HS. Transcription Factor NFAT5 Promotes Macrophage Survival in Rheumatoid Arthritis. J Clin Invest (2017) 127(3):954–69. doi: 10.1172/JCI87880 PMC5330733 28192374

[107] Li Y Zheng Y Li T Wang Q Qian J Lu Y. Chemokines CCL2, 3, 14 Stimulate Macrophage Bone Marrow Homing, Proliferation, and Polarization in Multiple Myeloma. Oncotarget (2015) 6(27):24218. doi: 10.18632/oncotarget.2965 26155942 PMC4695181

[108] Sierra-Filardi E Nieto C Domínguez-Soto Á Barroso R Sánchez-Mateos P Puig-Kroger A. CCL2 Shapes Macrophage Polarization by GM-CSF and M-CSF: Identification of CCL2/CCR2-Dependent Gene Expression Profile. J Immunol (2014) 192(8):3858–67. doi: 10.4049/jimmunol.1302821 24639350

[109] Nio Y Yamauchi T Iwabu M Okada-Iwabu M Funata M Yamaguchi M. Monocyte Chemoattractant Protein-1 (MCP-1) Deficiency Enhances Alternatively Activated M2 Macrophages and Ameliorates Insulin Resistance and Fatty Liver in Lipoatrophic Diabetic A-ZIP Transgenic Mice. Diabetologia (2012) 55(12):3350–8. doi: 10.1007/s00125-012-2710-2 22983634

[110] Lumeng CN Bodzin JL Saltiel AR. Obesity Induces a Phenotypic Switch in Adipose Tissue Macrophage Polarization. J Clin Invest (2007) 117(1):175–84. doi: 10.1172/JCI29881 PMC1716210 17200717

[111] Salcedo R Ponce ML Young HA Wasserman K Ward JM Kleinman HK. Human Endothelial Cells Express CCR2 and Respond to MCP-1: Direct Role of MCP-1 in Angiogenesis and Tumor Progression. Blood J Am Soc Hematol (2000) 96(1):34–40. 10891427

[112] Webster NL Crowe SM. Matrix Metalloproteinases, Their Production by Monocytes and Macrophages and Their Potential Role in HIV-Related Diseases. J Leukoc Biol (2006) 80(5):1052–66. doi: 10.1189/jlb.0306152 16959898

[113] Okuma T Terasaki Y Kaikita K Kobayashi H Kuziel WA Kawasuji M. C-C Chemokine Receptor 2 (CCR2) Deficiency Improves Bleomycin-Induced Pulmonary Fibrosis by Attenuation of Both Macrophage Infiltration and Production of Macrophage-Derived Matrix Metalloproteinases. J Pathol (2004) 204(5):594–604. doi: 10.1002/path.1667 15538737

[114] Carr MW Roth SJ Luther E Rose SS Springer TA. Monocyte Chemoattractant Protein 1 Acts as a T-Lymphocyte Chemoattractant. Proc Natl Acad Sci USA (1994) 91(9):3652–6. doi: 10.1073/pnas.91.9.3652 PMC43639 8170963

[115] Connor SJ Paraskevopoulos N Newman R Cuan N Hampartzoumian T Lloyd AR. CCR2 Expressing CD4+ T Lymphocytes Are Preferentially Recruited to the Ileum in Crohn's Disease. Gut (2004) 53(9):1287–94. doi: 10.1136/gut.2003.028225 PMC1774196 15306587

[116] Traynor TR Kuziel WA Toews GB Huffnagle GB. CCR2 Expression Determines T1 *Versus* T2 Polarization During Pulmonary Cryptococcus Neoformans Infection. J Immunol (2000) 164(4):2021–7. doi: 10.4049/jimmunol.164.4.2021 10657654

[117] Traynor TR Herring AC Dorf ME Kuziel WA Toews GB Huffnagle GB. Differential Roles of CC Chemokine Ligand 2/Monocyte Chemotactic Protein-1 and CCR2 in the Development of T1 Immunity. J Immunol (2002) 168(9):4659–66. doi: 10.4049/jimmunol.168.9.4659 11971015

[118] Zhang N Schroppel B Lal G Jakubzick C Mao X Chen D. Regulatory T Cells Sequentially Migrate From Inflamed Tissues to Draining Lymph Nodes to Suppress the Alloimmune Response. Immunity (2009) 30(3):458–69. doi: 10.1016/j.immuni.2008.12.022 PMC2737741 19303390

[119] Zhan Y Wang N Vasanthakumar A Zhang Y Chopin M Nutt SL. CCR2 Enhances CD25 Expression by FoxP3(+) Regulatory T Cells and Regulates Their Abundance Independently of Chemotaxis and CCR2(+) Myeloid Cells. Cell Mol Immunol (2020) 17(2):123–32. doi: 10.1038/s41423-018-0187-8 PMC7000403 30538272

[120] Vasanthakumar A Chisanga D Blume J Gloury R Britt K Henstridge DC. Sex-Specific Adipose Tissue Imprinting of Regulatory T Cells. Nature (2020) 579(7800):581–5. doi: 10.1038/s41586-020-2040-3 PMC7241647 32103173

[121] Trujillo JA Fleming EL Perlman S. Transgenic CCL2 Expression in the Central Nervous System Results in a Dysregulated Immune Response and Enhanced Lethality After Coronavirus Infection. J Virol (2013) 87(5):2376–89. doi: 10.1128/JVI.03089-12 PMC3571407 23269787

[122] Karpus WJ Lukacs NW Kennedy KJ Smith WS Hurst SD Barrett TA. Differential CC Chemokine-Induced Enhancement of T Helper Cell Cytokine Production. J Immunol (1997) 158(9):4129–36. 9126972

[123] Bakos E Thaiss CA Kramer MP Cohen S Radomir L Orr I. CCR2 Regulates the Immune Response by Modulating the Interconversion and Function of Effector and Regulatory T Cells. J Immunol (2017) 198(12):4659–71. doi: 10.4049/jimmunol.1601458 28507030

[124] Pandiyan P Zheng L Ishihara S Reed J Lenardo MJ. CD4+ CD25+ Foxp3+ Regulatory T Cells Induce Cytokine Deprivation–Mediated Apoptosis of Effector CD4+ T Cells. Nat Immunol (2007) 8(12):1353–62. doi: 10.1038/ni1536 17982458

[125] Von Luettichau I Segerer S Wechselberger A Notohamiprodjo M Nathrath M Kremer M. A Complex Pattern of Chemokine Receptor Expression Is Seen in Osteosarcoma. BMC Cancer (2008) 8(1):1–10. doi: 10.1186/1471-2407-8-23 18215331 PMC2257957

[126] Wyler L Napoli C Ingold B Sulser T Heikenwälder M Schraml P. Brain Metastasis in Renal Cancer Patients: Metastatic Pattern, Tumor-Associated Macrophages and Chemokine/Chemoreceptor Expression. Br J Cancer (2014) 110(3):686–94. doi: 10.1038/bjc.2013.755 PMC3915122 24327013

[127] Macanas-Pirard P Quezada T Navarrete L Broekhuizen R Leisewitz A Nervi B. The CCL2/CCR2 Axis Affects Transmigration and Proliferation But Not Resistance to Chemotherapy of Acute Myeloid Leukemia Cells. PloS One (2017) 12(1):e0168888. doi: 10.1371/journal.pone.0168888 28045930 PMC5207636

[128] Lu Y Cai Z Xiao G Liu Y Keller ET Yao Z. CCR2 Expression Correlates With Prostate Cancer Progression. J Cell Biochem (2007) 101(3):676–85. doi: 10.1002/jcb.21220 17216598

[129] Fang WB Jokar I Zou A Lambert D Dendukuri P Cheng N. CCL2/CCR2 Chemokine Signaling Coordinates Survival and Motility of Breast Cancer Cells Through Smad3 Protein- and P42/44 Mitogen-Activated Protein Kinase (MAPK)-Dependent Mechanisms. J Biol Chem (2012) 287(43):36593–608. doi: 10.1074/jbc.M112.365999 PMC3476325 22927430

[130] Mierke CT Zitterbart DP Kollmannsberger P Raupach C Schlötzer-Schrehardt U Goecke TW. Breakdown of the Endothelial Barrier Function in Tumor Cell Transmigration. Biophys J (2008) 94(7):2832–46. doi: 10.1529/biophysj.107.113613 PMC2267111 18096634

[131] Yao M Fang W Smart C Hu Q Huang S Alvarez N. CCR2 Chemokine Receptors Enhance Growth and Cell-Cycle Progression of Breast Cancer Cells Through SRC and PKC Activation. Mol Cancer Res (2019) 17(2):604–17. doi: 10.1158/1541-7786.MCR-18-0750 PMC6359961 30446625

[132] Fang WB Sofia Acevedo D Smart C Zinda B Alissa N Warren K. Expression of CCL2/CCR2 Signaling Proteins in Breast Carcinoma Cells Is Associated With Invasive Progression. Sci Rep (2021) 11(1):8708. doi: 10.1038/s41598-021-88229-0 33888841 PMC8062684

[133] Liu JF Chen PC Chang TM Hou CH. Monocyte Chemoattractant Protein-1 Promotes Cancer Cell Migration *via* C-Raf/MAPK/AP-1 Pathway and MMP-9 Production in Osteosarcoma. J Exp Clin Cancer Res (2020) 39(1):254. doi: 10.1186/s13046-020-01756-y 33228783 PMC7684958

[134] An J Xue Y Long M Zhang G Zhang J Su H. Targeting CCR2 With Its Antagonist Suppresses Viability, Motility and Invasion by Downregulating MMP-9 Expression in Non-Small Cell Lung Cancer Cells. Oncotarget (2017) 8(24):39230–40. doi: 10.18632/oncotarget.16837 PMC5503609 28424406

[135] Zhang X-w Qin X Qin CY Yin Y-l Chen Y Zhu H-l. Expression of Monocyte Chemoattractant Protein-1 and CC Chemokine Receptor 2 in Non-Small Cell Lung Cancer and Its Significance. Cancer Immunol Immunother (2013) 62(3):563–70. doi: 10.1007/s00262-012-1361-y PMC11028706 23090289

[136] Li X Yao W Yuan Y Chen P Li B Li J. Targeting of Tumor-Infiltrating Macrophages *via* CCL2/CCR2 Signaling as a Therapeutic Strategy Against Hepatocellular Carcinoma. Gut (2017) 66(1):157–67. doi: 10.1136/gutjnl-2015-310514 26452628

[137] Dagouassat M Suffee N Hlawaty H Haddad O Charni F Laguillier C. Monocyte Chemoattractant Protein-1 (MCP-1)/CCL2 Secreted by Hepatic Myofibroblasts Promotes Migration and Invasion of Human Hepatoma Cells. Int J Cancer (2010) 126(5):1095–108. doi: 10.1002/ijc.24800 19642141

[138] Li H Li H Li XP Zou H Liu L Liu W. C−C Chemokine Receptor Type 2 Promotes Epithelial−to−Mesenchymal Transition by Upregulating Matrix Metalloproteinase−2 in Human Liver Cancer. Oncol Rep (2018) 40(5):2734–41. doi: 10.3892/or.2018.6660 30132565

[139] Levina V Nolen BM Marrangoni AM Cheng P Marks JR Szczepanski MJ. Role of Eotaxin-1 Signaling in Ovarian Cancer. Clin Cancer Res (2009) 15(8):2647–56. doi: 10.1158/1078-0432.CCR-08-2024 PMC2669845 19351767

[140] Garibay-Cerdenares OL Hernández-Ramírez VI Osorio-Trujillo JC Gallardo-Rincón D Talamás-Rohana P. Haptoglobin and CCR2 Receptor Expression in Ovarian Cancer Cells That Were Exposed to Ascitic Fluid: Exploring a New Role of Haptoglobin in the Tumoral Microenvironment. Cell Adh Migr (2015) 9(5):394–405. doi: 10.1080/19336918.2015.1035504 26211665 PMC4955374

[141] Xu W Wei Q Han M Zhou B Wang H Zhang J. CCL2-SQSTM1 Positive Feedback Loop Suppresses Autophagy to Promote Chemoresistance in Gastric Cancer. Int J Biol Sci (2018) 14(9):1054–66. doi: 10.7150/ijbs.25349 PMC6036739 29989092

[142] Mytar B Stec M Szatanek R Węglarczyk K Szewczyk K Szczepanik A. Characterization of Human Gastric Adenocarcinoma Cell Lines Established From Peritoneal Ascites. Oncol Lett (2018) 15(4):4849–58. doi: 10.3892/ol.2018.7995 PMC5840753 29552124

[143] Li R Zhang H Liu H Lin C Cao Y Zhang W. High Expression of C-C Chemokine Receptor 2 Associates With Poor Overall Survival in Gastric Cancer Patients After Surgical Resection. Oncotarget (2016) 7(17):23909–18. doi: 10.18632/oncotarget.8069 PMC5029673 26992207

[144] Monti P Leone BE Marchesi F Balzano G Zerbi A Scaltrini F. The CC Chemokine MCP-1/CCL2 in Pancreatic Cancer Progression: Regulation of Expression and Potential Mechanisms of Antimalignant Activity. Cancer Res (2003) 63(21):7451–61. 14612545

[145] Sanford DE Belt BA Panni RZ Mayer A Deshpande AD Carpenter D. Inflammatory Monocyte Mobilization Decreases Patient Survival in Pancreatic Cancer: A Role for Targeting the CCL2/CCR2 Axis. Clin Cancer Res (2013) 19(13):3404–15. doi: 10.1158/1078-0432.CCR-13-0525 PMC3700620 23653148

[146] Küper C Beck FX Neuhofer W. Autocrine MCP-1/CCR2 Signaling Stimulates Proliferation and Migration of Renal Carcinoma Cells. Oncol Lett (2016) 12(3):2201–9. doi: 10.3892/ol.2016.4875 PMC4998526 27602164

[147] Wang Z Xie H Zhou L Liu Z Fu H Zhu Y. CCL2/CCR2 Axis Is Associated With Postoperative Survival and Recurrence of Patients With Non-Metastatic Clear-Cell Renal Cell Carcinoma. Oncotarget (2016) 7(32):51525–34. doi: 10.18632/oncotarget.10492 PMC5239494 27409666

[148] Samaniego R Estecha A Relloso M Longo N Escat JL Longo-Imedio I. Mesenchymal Contribution to Recruitment, Infiltration, and Positioning of Leukocytes in Human Melanoma Tissues. J Invest Dermatol (2013) 133(9):2255–64. doi: 10.1038/jid.2013.88 23446986

[149] Vergani E Di Guardo L Dugo M Rigoletto S Tragni G Ruggeri R. Overcoming Melanoma Resistance to Vemurafenib by Targeting CCL2-Induced miR-34a, miR-100 and miR-125b. Oncotarget (2016) 7(4):4428–41. doi: 10.18632/oncotarget.6599 PMC4826216 26684239

[150] Koellensperger E Gramley F Preisner F Leimer U Germann G Dexheimer V. Alterations of Gene Expression and Protein Synthesis in Co-Cultured Adipose Tissue-Derived Stem Cells and Squamous Cell-Carcinoma Cells: Consequences for Clinical Applications. Stem Cell Res Ther (2014) 5(3):65. doi: 10.1186/scrt454 24887580 PMC4076640

[151] Nalla AK Gogineni VR Gupta R Dinh DH Rao JS. Suppression of uPA and uPAR Blocks Radiation-Induced MCP-1 Mediated Recruitment of Endothelial Cells in Meningioma. Cell Signal (2011) 23(8):1299–310. doi: 10.1016/j.cellsig.2011.03.011 PMC3095686 21426933

[152] Yang Q Zhang J Zhang X Miao L Zhang W Jiang Z. C-C Motif Chemokine Ligand 2/C-C Receptor 2 Is Associated With Glioma Recurrence and Poor Survival. Exp Ther Med (2021) 21(6):564. doi: 10.3892/etm.2021.9996 33850536 PMC8027722

[153] Desbaillets I Tada M de Tribolet N Diserens AC Hamou MF Van Meir EG. Human Astrocytomas and Glioblastomas Express Monocyte Chemoattractant Protein-1 (MCP-1) *In Vivo* and In Vitro. Int J Cancer (1994) 58(2):240–7. doi: 10.1002/ijc.2910580216 7517920

[154] Ou B Cheng X Xu Z Chen C Shen X Zhao J. A Positive Feedback Loop of β-Catenin/CCR2 Axis Promotes Regorafenib Resistance in Colorectal Cancer. Cell Death Dis (2019) 10(9):643. doi: 10.1038/s41419-019-1906-5 31501414 PMC6733926

[155] Hu H Sun L Guo C Liu Q Zhou Z Peng L. Tumor Cell-Microenvironment Interaction Models Coupled With Clinical Validation Reveal CCL2 and SNCG as Two Predictors of Colorectal Cancer Hepatic Metastasis. Clin Cancer Res (2009) 15(17):5485–93. doi: 10.1158/1078-0432.CCR-08-2491 19706805

[156] Chun E Lavoie S Michaud M Gallini CA Kim J Soucy G. CCL2 Promotes Colorectal Carcinogenesis by Enhancing Polymorphonuclear Myeloid-Derived Suppressor Cell Population and Function. Cell Rep (2015) 12(2):244–57. doi: 10.1016/j.celrep.2015.06.024 PMC4620029 26146082

[157] Detering L Luehmann H Heo GS Liu Y Sultan D Reichert D. Targeted PET Imaging of Chemokine Receptor 2 in Head and Neck Cancer and Progression. J Nucl Med (2018) 59(supplement 1):1149–.

[158] Tsai WH Shih CH Lin CC Ho CK Hsu FC Hsu HC. Monocyte Chemotactic Protein-1 in the Migration of Differentiated Leukaemic Cells Toward Alveolar Epithelial Cells. Eur Respir J (2008) 31(5):957–62. doi: 10.1183/09031936.00135707 18216048

[159] Maffei M Funicello M Vottari T Gamucci O Costa M Lisi S. The Obesity and Inflammatory Marker Haptoglobin Attracts Monocytes *via* Interaction With Chemokine (C-C Motif) Receptor 2 (CCR2). BMC Biol (2009) 7:87. doi: 10.1186/1741-7007-7-87 20017911 PMC2809058

[160] Lu Y Cai Z Galson DL Xiao G Liu Y George DE. Monocyte Chemotactic Protein-1 (MCP-1) Acts as a Paracrine and Autocrine Factor for Prostate Cancer Growth and Invasion. Prostate (2006) 66(12):1311–8. doi: 10.1002/pros.20464 16705739

[161] Rhodes DR Yu J Shanker K Deshpande N Varambally R Ghosh D. ONCOMINE: A Cancer Microarray Database and Integrated Data-Mining Platform. Neoplasia (2004) 6(1):1–6. doi: 10.1016/S1476-5586(04)80047-2 15068665 PMC1635162

[162] Sahai E Astsaturov I Cukierman E DeNardo DG Egeblad M Evans RM. A Framework for Advancing Our Understanding of Cancer-Associated Fibroblasts. Nat Rev Cancer (2020) 20(3):174–86. doi: 10.1038/s41568-019-0238-1 PMC7046529 31980749

[163] Tsuyada A Chow A Wu J Somlo G Chu P Loera S. CCL2 Mediates Cross-Talk Between Cancer Cells and Stromal Fibroblasts That Regulates Breast Cancer Stem Cells. Cancer Res (2012) 72(11):2768–79. doi: 10.1158/0008-5472.CAN-11-3567 PMC3367125 22472119

[164] Yang X Lin Y Shi Y Li B Liu W Yin W. FAP Promotes Immunosuppression by Cancer-Associated Fibroblasts in the Tumor Microenvironment *via* STAT3-CCL2 Signaling. Cancer Res (2016) 76(14):4124–35. doi: 10.1158/0008-5472.CAN-15-2973 27216177

[165] Zhang J Chen L Xiao M Wang C Qin Z. FSP1+ Fibroblasts Promote Skin Carcinogenesis by Maintaining MCP-1-Mediated Macrophage Infiltration and Chronic Inflammation. Am J Pathol (2011) 178(1):382–90. doi: 10.1016/j.ajpath.2010.11.017 PMC3070559 21224075

[166] Lin ZY Chuang YH Chuang WL. Cancer-Associated Fibroblasts Up-Regulate CCL2, CCL26, IL6 and LOXL2 Genes Related to Promotion of Cancer Progression in Hepatocellular Carcinoma Cells. BioMed Pharmacother (2012) 66(7):525–9. doi: 10.1016/j.biopha.2012.02.001 22739041

[167] Li S Lu J Chen Y Xiong N Li L Zhang J. MCP-1-Induced ERK/GSK-3β/Snail Signaling Facilitates the Epithelial-Mesenchymal Transition and Promotes the Migration of MCF-7 Human Breast Carcinoma Cells. Cell Mol Immunol (2017) 14(7):621–30. doi: 10.1038/cmi.2015.106 PMC5520413 26996066

[168] Kleiner DE Stetler-Stevenson WG. Matrix Metalloproteinases and Metastasis. Cancer Chemother Pharmacol (1999) 43 Suppl:S42–51. doi: 10.1007/s002800051097 10357558

[169] Han R Gu S Zhang Y Luo A Jing X Zhao L. Estrogen Promotes Progression of Hormone-Dependent Breast Cancer Through CCL2-CCR2 Axis by Upregulation of Twist *via* PI3K/AKT/NF-κb Signaling. Sci Rep (2018) 8(1):1–13. doi: 10.1038/s41598-018-27810-6 29934505 PMC6015029

[170] Fein MR He X-Y Almeida AS Bružas E Pommier A Yan R. Cancer Cell CCR2 Orchestrates Suppression of the Adaptive Immune Response. J Exp Med (2020) 217(10). doi: 10.1084/jem.20181551 PMC7537399 32667673

[171] Rafei M Deng J Boivin M-N Williams P Matulis SM Yuan S. A MCP1 Fusokine With CCR2-Specific Tumoricidal Activity. Mol Cancer (2011) 10(1):121. doi: 10.1186/1476-4598-10-121 21943176 PMC3189909

[172] Li M-Q Li H-P Meng Y-H Wang X-Q Zhu X-Y Mei J. Chemokine CCL2 Enhances Survival and Invasiveness of Endometrial Stromal Cells in an Autocrine Manner by Activating Akt and MAPK/Erk1/2 Signal Pathway. Fertil steril (2012) 97(4):919–29.e1. doi: 10.1016/j.fertnstert.2011.12.049 22265030

[173] Xia M Sui Z. Recent Developments in CCR2 Antagonists. Expert Opin Ther patents (2009) 19(3):295–303. doi: 10.1517/13543770902755129 19441905

[174] Zhao Q Murtaza A Bata A Sun W Ho C-P Vuppugalla R. Abstract 3760: Preclinical Antitumor Activity of a CC Chemokine Receptor (CCR) 2/5 Dual Antagonist as Monotherapy and in Combination With Immune Checkpoint Blockade. Cancer Res (2018) 78(13 Supplement):3760. doi: 10.1158/1538-7445.AM2018-3760

[175] de Zeeuw D Bekker P Henkel E Hasslacher C Gouni-Berthold I Mehling H. The Effect of CCR2 Inhibitor CCX140-B on Residual Albuminuria in Patients With Type 2 Diabetes and Nephropathy: A Randomised Trial. Lancet Diabetes Endocrinol (2015) 3(9):687–96. doi: 10.1016/S2213-8587(15)00261-2 26268910

[176] Thompson M Saag M DeJesus E Gathe J Lalezari J Landay AL. A 48-Week Randomized Phase 2b Study Evaluating Cenicriviroc *Versus* Efavirenz in Treatment-Naive HIV-Infected Adults With C-C Chemokine Receptor Type 5-Tropic Virus. Aids (2016) 30(6):869–78. doi: 10.1097/QAD.0000000000000988 PMC4794136 26636929

[177] Bachelerie F Ben-Baruch A Burkhardt AM Combadiere C Farber JM Graham GJ. International Union of Basic and Clinical Pharmacology. LXXXIX. Update on the Extended Family of Chemokine Receptors and Introducing a New Nomenclature for Atypical Chemokine Receptors. Pharmacol Rev (2014) 66(1):1–79. doi: 10.1124/pr.113.007724 24218476 PMC3880466

[178] Brodmerkel CM Huber R Covington M Diamond S Hall L Collins R. Discovery and Pharmacological Characterization of a Novel Rodent-Active CCR2 Antagonist, Incb3344. J Immunol (2005) 175(8):5370–8. doi: 10.4049/jimmunol.175.8.5370 16210643

[179] Xue CB Feng H Cao G Huang T Glenn J Anand R. Discovery of INCB3284, A Potent, Selective, and Orally Bioavailable Hccr2 Antagonist. ACS Med Chem Lett (2011) 2(6):450–4. doi: 10.1021/ml200030q PMC4018154 24900329

[180] Zheng C Cao G Xia M Feng H Glenn J Anand R. Discovery of INCB10820/PF-4178903, a Potent, Selective, and Orally Bioavailable Dual CCR2 and CCR5 Antagonist. Bioorg Med Chem Lett (2011) 21(5):1442–6. doi: 10.1016/j.bmcl.2011.01.015 21295478

[181] Buntinx M Hermans B Goossens J Moechars D Gilissen RA Doyon J. Pharmacological Profile of JNJ-27141491 [(S)-3-[3,4-Difluorophenyl)-Propyl]-5-Isoxazol-5-Yl-2-Thioxo-2,3-Dihydro-1H-Imidazole-4-Carboxyl Acid Methyl Ester], as a Noncompetitive and Orally Active Antagonist of the Human Chemokine Receptor CCR2. J Pharmacol Exp Ther (2008) 327(1):1–9. doi: 10.1124/jpet.108.140723 18599682

[182] Noda M Tomonaga D Kitazono K Yoshioka Y Liu J Rousseau JP. Neuropathic Pain Inhibitor, RAP-103, Is a Potent Inhibitor of Microglial CCL1/CCR8. Neurochem Int (2018) 119:184–9. doi: 10.1016/j.neuint.2017.12.005 29248693

[183] Sayyed SG Ryu M Kulkarni OP Schmid H Lichtnekert J Grüner S. An Orally Active Chemokine Receptor CCR2 Antagonist Prevents Glomerulosclerosis and Renal Failure in Type 2 Diabetes. Kidney Int (2011) 80(1):68–78. doi: 10.1038/ki.2011.102 21508925

[184] Wang J Yunfu L Ba Y Guo F Xu D Li J. WXSH0213, a Dual CCR2 and CCR5 Antagonist, Ameliorates Nonalcoholic Activity Score and Liver Fibrosis MCD Mice. In: The 68th Annual Meeting of the American Association for the Study of Lover Duseases: The Liver Meeting 2017. (2017). 66:149–1185. doi: 10.1002/hep.29501

[185] Baeck C Wei X Bartneck M Fech V Heymann F Gassler N. Pharmacological Inhibition of the Chemokine C-C Motif Chemokine Ligand 2 (Monocyte Chemoattractant Protein 1) Accelerates Liver Fibrosis Regression by Suppressing Ly-6C(+) Macrophage Infiltration in Mice. Hepatology (2014) 59(3):1060–72. doi: 10.1002/hep.26783 24481979

[186] Huang D Gray JD Lu G Phillips W Carmody L Gastwirt R. Efficacy of Functionally Blocking Antibodies Against CC Chemokine Receptor 2 (CCR2). http://sorrentotherapeutics.com/wp-content/uploads/2013/10/Sorrento_Poster_Efficacy_of_Functionally_Blocking_Antibodies_Against_CCR2.pdf

[187] Astellas DM. KaliVir to Jointly Develop Oncolytic Virus VET2-L2 FirstWorld Pharma2020 . Available at: https://www.firstwordpharma.com/node/1780793.

[188] Haringman JJ Gerlag DM Smeets TJ Baeten D van den Bosch F Bresnihan B. A Randomized Controlled Trial With an Anti-CCL2 (Anti-Monocyte Chemotactic Protein 1) Monoclonal Antibody in Patients With Rheumatoid Arthritis. Arthritis Rheum (2006) 54(8):2387–92. doi: 10.1002/art.21975 16869001

[189] Brana I Calles A LoRusso PM Yee LK Puchalski TA Seetharam S. Carlumab, an Anti-CC Chemokine Ligand 2 Monoclonal Antibody, in Combination With Four Chemotherapy Regimens for the Treatment of Patients With Solid Tumors: An Open-Label, Multicenter Phase 1b Study. Targeted Oncol (2015) 10(1):111–23. doi: 10.1007/s11523-014-0320-2 24928772

[190] Chen X Wang Y Nelson D Tian S Mulvey E Patel B. CCL2/CCR2 Regulates the Tumor Microenvironment in HER-2/Neu-Driven Mammary Carcinomas in Mice. PloS One (2016) 11(11):e0165595. doi: 10.1371/journal.pone.0165595 27820834 PMC5098736

[191] Laviron M Boissonnas A. Ontogeny of Tumor-Associated Macrophages. Front Immunol (2019) 10:1799. doi: 10.3389/fimmu.2019.01799 31417566 PMC6684758

[192] Fritz JM Tennis MA Orlicky DJ Yin H Ju C Redente EF. Depletion of Tumor-Associated Macrophages Slows the Growth of Chemically Induced Mouse Lung Adenocarcinomas. Front Immunol (2014) 5:587. doi: 10.3389/fimmu.2014.00587 25505466 PMC4243558

[193] Mack M Cihak J Simonis C Luckow B Proudfoot AE Jí Plachý. Expression and Characterization of the Chemokine Receptors CCR2 and CCR5 in Mice. J Immunol (2001) 166(7):4697–704. doi: 10.4049/jimmunol.166.7.4697 11254730

[194] Klaver EJ Kuijk LM Lindhorst TK Cummings RD van Die I. Schistosoma Mansoni Soluble Egg Antigens Induce Expression of the Negative Regulators SOCS1 and SHP1 in Human Dendritic Cells *via* Interaction With the Mannose Receptor. PloS One (2015) 10(4):e0124089. doi: 10.1371/journal.pone.0124089 25897665 PMC4405200

[195] Deligne C Milcent B Josseaume N Teillaud J-L Siberil S. Impact of Depleting Therapeutic Monoclonal Antibodies on the Host Adaptive Immunity: A Bonus or a Malus? Front Immunol (2017) 8:950. doi: 10.3389/fimmu.2017.00950 28855903 PMC5557783

[196] Izumi Y Kanayama M Shen Z Kai M Kawamura S Akiyama M. An Antibody-Drug Conjugate That Selectively Targets Human Monocyte Progenitors for Anti-Cancer Therapy. Front Immunol (2021) 12:618081. doi: 10.3389/fimmu.2021.618081 33692791 PMC7937628

[197] Tada Y Togashi Y Kotani D Kuwata T Sato E Kawazoe A. Targeting VEGFR2 With Ramucirumab Strongly Impacts Effector/Activated Regulatory T Cells and CD8+ T Cells in the Tumor Microenvironment. J immunother Cancer (2018) 6(1):1–14. doi: 10.1186/s40425-018-0403-1 30314524 PMC6186121

[198] Shimizu J Yamazaki S Sakaguchi S. Induction of Tumor Immunity by Removing CD25+ CD4+ T Cells: A Common Basis Between Tumor Immunity and Autoimmunity. J Immunol (1999) 163(10):5211–8. 10553041

[199] Mondini M Loyher P-L Hamon P De Thoré MG Laviron M Berthelot K. CCR2-Dependent Recruitment of Tregs and Monocytes Following Radiotherapy Is Associated With Tnfα-Mediated Resistance. Cancer Immunol Res (2019) 7(3):376–87. doi: 10.1158/2326-6066.CIR-18-0633 30696630

[200] Nywening TM Wang-Gillam A Sanford DE Belt BA Panni RZ Cusworth BM. Phase 1b Study Targeting Tumor Associated Macrophages With CCR2 Inhibition Plus FOLFIRINOX in Locally Advanced and Borderline Resectable Pancreatic Cancer. Lancet Oncol (2016) 17(5):651. doi: 10.1016/S1470-2045(16)00078-4 27055731 PMC5407285

[201] Lim SY Yuzhalin AE Gordon-Weeks AN Muschel RJ. Targeting the CCL2-CCR2 Signaling Axis in Cancer Metastasis. Oncotarget (2016) 7(19):28697. doi: 10.18632/oncotarget.7376 26885690 PMC5053756

[202] Sun W Li W-J Wei F-Q Wong T-S Lei W-B Zhu X-L. Blockade of MCP-1/CCR4 Signaling-Induced Recruitment of Activated Regulatory Cells Evokes an Antitumor Immune Response in Head and Neck Squamous Cell Carcinoma. Oncotarget (2016) 7(25):37714. doi: 10.18632/oncotarget.9265 27177223 PMC5122343

[203] Zhang T Somasundaram R Berencsi K Caputo L Gimotty P Rani P. Migration of Cytotoxic T Lymphocytes Toward Melanoma Cells in Three-Dimensional Organotypic Culture Is Dependent on CCL2 and CCR4. Eur J Immunol (2006) 36(2):457–67. doi: 10.1002/eji.200526208 16421945

[204] Liu Y Cai Y Liu L Wu Y Xiong X. Crucial Biological Functions of CCL7 in Cancer. PeerJ (2018) 6:e4928. doi: 10.7717/peerj.4928 29915688 PMC6004300

[205] Kitamura T Qian B-Z Soong D Cassetta L Noy R Sugano G. CCL2-Induced Chemokine Cascade Promotes Breast Cancer Metastasis by Enhancing Retention of Metastasis-Associated Macrophages. J Exp Med (2015) 212(7):1043–59. doi: 10.1084/jem.20141836 PMC4493415 26056232

[206] Zhao L Zhang F Li L Chen S Hu Y. Computational Design of Novel Tetra-Specific Antibody (PD-1/CD47/VEGF/TGF-ß) With IgG-Like Architecture Against Non-Small Cell Lung Cancer (NSCLC). Am Soc Clin Oncol (2019) 37(15_suppl):e14002. doi: 10.1200/JCO.2019.37.15_suppl.e14002

[207] Lu X Kang Y. Chemokine (CC Motif) Ligand 2 Engages CCR2+ Stromal Cells of Monocytic Origin to Promote Breast Cancer Metastasis to Lung and Bone. J Biol Chem (2009) 284(42):29087–96. doi: 10.1074/jbc.M109.035899 PMC2781454 19720836

[208] Murthy V Minehart J Sterman DH. Local Immunotherapy of Cancer: Innovative Approaches to Harnessing Tumor-Specific Immune Responses. J Natl Cancer Inst (2017) 109(12). doi: 10.1093/jnci/djx097 29546344

[209] Yu P Lee Y Liu W Krausz T Chong A Schreiber H. Intratumor Depletion of CD4+ Cells Unmasks Tumor Immunogenicity Leading to the Rejection of Late-Stage Tumors. J Exp Med (2005) 201(5):779–91. doi: 10.1084/jem.20041684 PMC2212829 15753211

[210] Slegers TP van der Gaag R van Rooijen N van Rij G Streilein JW. Effect of Local Macrophage Depletion on Cellular Immunity and Tolerance Evoked by Corneal Allografts. Curr Eye Res (2003) 26(2):73–9. doi: 10.1076/ceyr.26.2.73.14510 12815525

[211] Guillamat-Prats R CamprubÌ-Rimblas M Tantiny N Artigas A. Local Depletion of the Recruitment of Macrophages Reduces Acute Lung Injury in Rats. Eur Respir J (2018) 52. doi: 10.1183/13993003.congress-2018.PA4296

[212] Cignetti A Vallario A Roato I Circosta P Strola G Scielzo C. The Characterization of Chemokine Production and Chemokine Receptor Expression Reveals Possible Functional Cross-Talks in AML Blasts With Monocytic Differentiation. Exp Hematol (2003) 31(6):495–503. doi: 10.1016/S0301-472X(03)00066-3 12829025

[213] Jacamo RO Mu H Zhang Q Chachad D Zhiqiang W Ma W. Effects of CCL2/CCR2 Blockade in Acute Myeloid Leukemia. Blood (2015) 126(23):1348. doi: 10.1182/blood.V126.23.1348.1348

[214] Corcione A Tortolina G Bonecchi R Battilana N Taborelli G Malavasi F. Chemotaxis of Human Tonsil B Lymphocytes to CC Chemokine Receptor (CCR) 1, CCR2 and CCR4 Ligands Is Restricted to Non-Germinal Center Cells. Int Immunol (2002) 14(8):883–92. doi: 10.1093/intimm/dxf054 12147625

[215] Trentin L Cabrelle A Facco M Carollo D Miorin M Tosoni A. Homeostatic Chemokines Drive Migration of Malignant B Cells in Patients With Non-Hodgkin Lymphomas. Blood (2004) 104(2):502–8. doi: 10.1182/blood-2003-09-3103 15001469

[216] van Attekum MH van Bruggen JA Slinger E Lebre MC Reinen E Kersting S. CD40 Signaling Instructs Chronic Lymphocytic Leukemia Cells to Attract Monocytes *via* the CCR2 Axis. haematologica (2017) 102(12):2069. doi: 10.3324/haematol.2016.157206 28971904 PMC5709106

[217] Tu MM Abdel-Hafiz HA Jones RT Jean A Hoff KJ Duex JE. Inhibition of the CCL2 Receptor, CCR2, Enhances Tumor Response to Immune Checkpoint Therapy. Commun Biol (2020) 3(1):720. doi: 10.1038/s42003-020-01441-y 33247183 PMC7699641

[218] Wu X Singh R Hsu DK Zhou Y Yu S Han D. A Small Molecule CCR2 Antagonist Depletes Tumor Macrophages and Synergizes With Anti–PD-1 in a Murine Model of Cutaneous T-Cell Lymphoma (CTCL). J Invest Dermatol (2020) 140(7):1390–400. e4. doi: 10.1016/j.jid.2019.11.018 31945344

[219] Hegde S Krisnawan VE Herzog BH Zuo C Breden MA Knolhoff BL. Dendritic Cell Paucity Leads to Dysfunctional Immune Surveillance in Pancreatic Cancer. Cancer Cell (2020) 37(3):289–307.e9. doi: 10.1016/j.ccell.2020.02.008 32183949 PMC7181337

[220] Nakasone ES Askautrud HA Kees T Park J-H Plaks V Ewald AJ. Imaging Tumor-Stroma Interactions During Chemotherapy Reveals Contributions of the Microenvironment to Resistance. Cancer Cell (2012) 21(4):488–503. doi: 10.1016/j.ccr.2012.02.017 22516258 PMC3332002

[221] Brana I Calles A LoRusso PM Yee LK Puchalski TA Seetharam S. Carlumab, an Anti-C-C Chemokine Ligand 2 Monoclonal Antibody, in Combination With Four Chemotherapy Regimens for the Treatment of Patients With Solid Tumors: An Open-Label, Multicenter Phase 1b Study. Target Oncol (2015) 10(1):111–23. doi: 10.1007/s11523-014-0320-2 24928772

[222] Connolly KA Belt BA Figueroa NM Murthy A Patel A Kim M. Increasing the Efficacy of Radiotherapy by Modulating the CCR2/CCR5 Chemokine Axes. Oncotarget (2016) 7(52):86522. doi: 10.18632/oncotarget.13287 27852031 PMC5349932

[223] Sandhu SK Papadopoulos K Fong PC Patnaik A Messiou C Olmos D. A First-in-Human, First-in-Class, Phase I Study of Carlumab (CNTO 888), a Human Monoclonal Antibody Against CC-Chemokine Ligand 2 in Patients With Solid Tumors. Cancer Chemother Pharmacol (2013) 71(4):1041–50. doi: 10.1007/s00280-013-2099-8 23385782

[224] Pienta KJ Machiels J-P Schrijvers D Alekseev B Shkolnik M Crabb SJ. Phase 2 Study of Carlumab (CNTO 888), A Human Monoclonal Antibody Against CC-Chemokine Ligand 2 (CCL2), in Metastatic Castration-Resistant Prostate Cancer. Invest New Drugs (2013) 31(3):760–8. doi: 10.1007/s10637-012-9869-8 22907596

